# A 28-day, 2-year study reveals that adolescents are more fatigued and distressed on days with greater NO_2_ and CO air pollution

**DOI:** 10.1038/s41598-022-20602-z

**Published:** 2022-10-11

**Authors:** Emma Armstrong-Carter, Andrew J. Fuligni, Xiao Wu, Nancy Gonzales, Eva H. Telzer

**Affiliations:** 1 University of California, Berkeley, Berkeley, CA, USA; 2 Stanford University, Stanford, CA USA; 3 University of North Carolina at Chapel Hill, Chapel Hill, NC, USA; 4 University of California, Los Angeles, Los Angeles, CA, USA; 5 Arizona State University, Tempe, AZ, USA

**Keywords:** Human behaviour, Environmental sciences, Biomarkers

## Abstract

This 2-year, 28-day study examined whether adolescents felt greater fatigue and emotional distress the same day and the day after air quality was worse. We linked objective daily air quality measurements to daily self-reports from 422 Mexican–American adolescents in Los Angeles County, California from 2009 to 2011 (50% girls, M_*Age*_ = 15 years). A robust, within-subject analysis of 9696 observations revealed that adolescents with ongoing physical complaints reported greater fatigue and emotional distress on days that the air contained higher levels of nitrogen dioxide (NO_2_) and carbon monoxide (CO). Regardless of physical complaints, adolescents on average also reported greater fatigue the day after NO_2_ levels were higher. The same-day and next-day associations between air pollution and distress were mediated via daily increases in fatigue. Results were robust when controlling for day of the week, and daily temperature and humidity. Sulfur dioxide (SO_2_), ozone (O_3_), PM_2.5_ and PM_10_ were not related to daily fatigue or distress.

## Introduction

Air pollution is one of the biggest environmental threats to human health worldwide. Every year, exposure to air pollution results in the loss of millions of healthy years of life^1^. Numerous studies have found adverse effects of air pollution on adolescents’ health, focusing mainly on physical conditions such as respiratory symptoms^2–4^. In addition, a few studies have shown that youth who are exposed to worse air quality experience greater fatigue and emotional problems at the time of exposure and later in life^5–9^. However, prior research has focused on between-subject comparisons^10^, so these findings may be confounded by many individual differences between youth. It remains unknown if adolescents’ experiences of fatigue and emotional distress fluctuate on a daily level with air quality, within individuals. Such knowledge can provide greater temporal specificity for understanding air pollution as a risk-factor for youths’ emerging emotional difficulties. This within-subject, daily-level study investigated whether adolescents experienced greater fatigue and emotional distress on days that air quality was degraded in LA county, when controlling for between-subject effects. Further, we examined whether air quality was related on a daily level to fatigue and distress particularly strongly among adolescents who experienced greater physical symptoms throughout the year (e.g., headache, backpain). Finally, we considered alternative specifications such as lagged effects, and indirect pathways to shed light on potential directionality.

### Air pollution and adolescents’ mood

Air pollution includes gaseous pollutants that circulate in the air—such as nitrogen dioxide (NO_2_), carbon monoxide (CO), and sulfur dioxide (SO_2_). These pollutants are emitted from transportation vehicles, coal-driven thermal power plants, industries, indoor heating, and cooking^11^. Ozone (O_3_), secondary gaseous pollutant that is formed in the lower atmosphere by chemical reactions, also contributes to air pollution. Although air pollution decreased in the United States (US) from the 1980s until 2016 due to the Clean Air Act (42 US Code 7408-9) which regulated emissions^11^, air pollution has since increased^12^.

Adolescence is a sensitive growth period characterized by rapid biological, neurological, and social changes that contribute to heightened susceptibility for the emergence of emotional challenges^13^. For example, adolescents undergo changes associated with puberty, physical growth, circadian rhythms, psychopathology and daily mood reactivity, all of which impact their cognitive, emotional, social and motivational processes^13^. Adolescents’ emotional wellbeing is related strongly to their physical surroundings and environments^14^. Many cross-sectional and longitudinal studies in Asia indicate that youth who are exposed to higher levels of air pollution report greater emotional disturbances such as anxiety and stress^8,15^. Similarly, studies in the global West have shown that adolescents who are exposed to greater air pollution on average show experience more emotional challenges^5,6,9^. For example, children in the UK who were lived in greater air pollution at age 10 -12 were more likely to develop depressive symptoms at age 18^6^. This work converges with meta-analytical findings and suggests that exposure to air pollution is a risk factor for adolescents’ emotional health problems^10^.

The majority of studies linking air quality to fatigue and emotional health have used between-subject analyses^10^. Although informative, between-subjects analyses offer limited insight because they do not account for many individual differences between youth, even when covariates are included^16^. Relevant individual differences for the study of air quality and wellbeing include features of the youth’s environment, family life, school life, underlying physiological response patterns, and individual risk for physical and emotional challenges^10^. For example, families with low socio-economic status tend to live in the more polluted areas^10^, so adolescents’ emotional challenges could be associated with the underlying financial and social difficulties, instead of air pollution uniquely. To mitigate the confounding from between-subject designs, within-subjects analyses can clarify the link between air pollution and adolescents’ mood by treating individual youth as their own controls, and comparing their experiences and exposures on 1 day to other days. Within-subjects analyses also offer greater temporal specificity, because air pollution may be associated with adolescents’ mood on a daily level, for example within 1 or 2 days. This robust approach requires assessments collected from the same individuals repeatedly over time. Although methodologically intensive, such work can clarify whether adolescents face heightened risk for fatigue or emotional challenges on days—or the day after—they are exposed to greater pollution.

A few studies, mostly from Asia, have demonstrated significant daily-level associations between air quality and human physical and emotional health. In India and China, hospital admissions for asthma and airway obstructions increased on days that air quality was worse^17^ and on days after air quality was worse the previous day^18^, even among children^19^. Similarly, mortality increased on days after air quality was worse in Canada^20^. Two studies extended this work on physical health to examine daily links between air pollution and adults’ emotional health^21,22^. In China, adults’ hospital admissions for emotional health problems (e.g., schizophrenia, depression) increased on days that air quality was worse, particularly when levels of NO_2_ were higher^21^, and also 7 days later^22^. However, no known studies have examined daily links between air quality and adolescents’ emotional health, or have addressed daily links between air quality and emotional health in the US context. Given these within-subject, daily links between pollution and adults’ emotional health^21,22^, and also the previous between-subject, cross-sectional links between pollution and youths’ emotional health^19^, it is highly likely that pollution is also associated within-subjects on a daily level to adolescents’ emotional health. It is important to extending prior within-subjects research on this topic to the adolescent period, because adolescence is a sensitive developmental transition for the emergence emotional difficulties^13^. Such work can facilitate and understanding of how to support adolescents’ emotional wellbeing.

### Potential mechanisms linking air pollution to mood

Air pollution may impact adolescents’ mood via several biological pathways. One pathway is inflammation, which taxes the body physiologically and can contribute to feelings of fatigue. Air pollution increases humans’ levels of circulating inflammation in the blood, such as C-reactive protein^23^. In turn, inflammation is robustly linked to heightened symptoms of exhaustion, anxiety and depression in both adolescents and adults^24^. Specifically, inflammation impacts both the body and brain via multiple different neuroendocrinological and immunological mechanisms that occur simultaneously. For instance, inflammation increases monoamine levels, contributes to dysregulation of the hypothalamic–pituitary–adrenal axis, and activates pathologic microglial cells^23^. All of these biological pathways are physiologically arduous for the body and can contribute to feelings of fatigue. Moreover, exposure to air pollution also impairs the immunological, neuroendocrine and autonomic pathways which promote healthy physiological homeostasis, rest and sleep^25^, which can further contribute to fatigue. Accordingly, pollution may “get under the skin” as inflammatory processes that contribute to adolescents’ fatigue by taxing the body and reducing adolescents’ ability to maintain homeostasis and rest adequately^23^. In turn, fatigue reduces adolescents’ capacity to effectively self-regulate difficult emotions and maintain emotional wellbeing throughout the day^26^. This research suggests that air pollution may impact adolescents’ mood via an indirect pathway by which youth become more fatigued, which in turn leads to greater emotional problems such as anxiety and depressive symptoms that day or even the following day.

### Youth with physical symptoms may be more sensitive to air pollution

Air pollution does not impact adolescents’ moods uniformly. Adolescents who experience higher levels of ongoing physical symptoms (e.g., headaches, stomachaches, shortness of breath) may be particularly sensitive to the daily effects of air pollution on fatigue and emotional health^1^. Compromised physical health can make individuals more susceptible to physiological stress from the environment^27^. Further, ongoing physical symptoms associated with health challenges or illness are linked bidirectionally with depression, neuroendocrine dysregulation, and inflammation^28^. Adolescents who experience greater ongoing physical symptoms face higher risk for emotional problems, and also likely suffer underlying cardiometabolic risks that make their body more susceptible to impairments caused by air pollution (e.g., inflammation, poor sleep, neuroendocrine changes)^29^. In addition, adolescents who experience more physical symptoms have more compromised immune systems^30^, which can hinder their ability to ward off and cope with the negative physiological effects of air pollution. For example, children with asthma were more vulnerable to the additional negative physical health impacts of air pollution compared to their non-asthmatic peers^31,32^. Accordingly, it is likely that air pollution is associated on a daily level with feelings of fatigue and emotional distress more strongly among adolescents who experience relatively higher levels of ongoing physical symptoms compared to their peers.

### Current study

The goal of this study was to understand if air pollution is related on a daily level to adolescents’ experiences of fatigue and emotional distress. We linked adolescents’ daily reports of fatigue and emotional distress to objective daily, county-level air pollution data collected by the EPA in LA County from 2009 to 2011^33^. All participants lived in LA County at the time they completed daily diaries. We investigated the following questions: (1) When air pollution is higher, do adolescents feel more fatigue and emotional distress the same day and the following day? We operationalized emotional distress as experiences of anxiety, sadness and stress. For air quality, we focused on four major criteria-gas air pollutants regulated by the Clean Air Act: NO_2_, CO, O_3_, and SO_2_^33^. We hypothesized that when air pollution was higher, adolescents would report greater fatigue and emotional distress the same day and the next day. (2) Is air quality related to adolescents’ fatigue and emotional distress the same day and the next day more strongly among those who report higher levels of ongoing physical symptoms that year? We hypothesized that air pollution would be more strongly associated on a daily level with fatigue and distress among adolescents who reported relatively higher levels of physical symptoms that year compared to their peers. (3) Finally, are the daily-level links between air pollution and distress mediated via an indirect pathway through daily levels of fatigue? We hypothesized that daily levels of fatigue would significantly mediate the daily, positive link between air pollution and emotional distress. Since these processes likely occur both within and across days, we tested both same-day and 1-day-lagged mediation models. In addition to these primary analyses, we tested alternative specifications as detailed further below.

## Results

### Descriptive statistics and correlations

The Supplementary Materials display full descriptive information (Table S1) and bivariate correlations (Table S2), as well as more information about pollutant-specific Air Quality Indices (AQIs), which we used for measuring air pollution. During the study days, there were low levels of CO (*M* = 6.32, *SD* = 3.56, *Range* = 1.93–20.50) and SO_2_ (*M* = 2.57, *SD* = 2.29, *Range* = 1.00–17.25) and all daily observations fell within the “healthy” category defined by the EPA^33^. There were relatively higher levels of NO_2_ (*M* = 28.71, *SD* = 9.97, *Range* = 7.27–60.33), and O_3_ (*M* = 42.60, *SD* = 16.89, *Range* = 8.23–114.13), but most (72.22%) of the daily observations still fell within the “healthy” category (98% for NO_2_ and 72% for O_3_). Adolescents reported moderate levels of daily fatigue (*M* = 1.71, *SD* = 0.89, *Range* = 1–5), daily emotional distress (*M* = 1.51, *SD* = 0.69, *Range* = 1–5), and yearly physical symptoms (*M* = 1.58, *SD* = 0.45, *Range* = 1–3.58). On average across all days of the study, all pollutants were correlated with each other (*p* < 0.05), but not with fatigue, emotional distress, or physical symptoms (*p* > 0.05).

### Same-day multilevel regressions

O_3_ and SO_2_ were not related significantly to adolescents’ daily levels of fatigue or distress, so those results displayed in the Supplementary Materials (Table S3). As shown in Table 1, Model 1, on the average, between-subjects level, adolescents who were exposed to higher levels of NO_2_ and CO and experienced more ongoing physical symptoms over the course of two weeks, and reported higher levels of fatigue and emotional distress. On the daily, within-subjects level, adolescents felt higher levels of fatigue and emotional distress on days that NO_2_ levels were higher in the air. However, this daily association was qualified by a significant interaction in Model 2.

**Table 1:** Daily and average level associations between air pollutants NO_2_ and CO predicting adolescents′ fatigue and distress, using three level hierarchical linear regression models that nested days within years within individuals. Weekday was coded 1 = weekday 2 = not a weekday.

	Daily Fatigue				Daily Emotional Distress			
	NO_2_ → Fatigue		CO → Fatigue		NO_2_ → Distress		CO → Distress	
	Model 1	Model 2	Model 1	Model 2	Model 1	Model 2	Model 1	Model 2
	*B*	*B*	*B*	*B*	*B*	*B*	*B*	*B*
	*(SE)*	*(SE)*	*(SE)*	*(SE)*	*(SE)*	*(SE)*	*(SE)*	*(SE)*
Weekday	**0.056**^**a**^	**0.053**^**a**^	**0.073**^**a**^	**0.073**^**a**^	**0.071**^**a**^	**0.071**^**a**^	**0.076**^**a**^	**0.077**^**a**^
	(0.015)	(0.015)	(0.015)	(0.015)	(0.011)	(0.011)	(0.010)	(0.010)
Person Mean-Centered Daily Pollutant level	**0.004**^**a**^	**0.004**^**a**^	0.006	0.006	**0.001**^**c**^	**0.001**^**c**^	0.004	0.004
	(0.001)	(0.001)	(0.003)	(0.003)	(0.001)	(0.001)	(0.002)	(0.002)
Person Average Pollutant Level	**0.009**^**c**^	0.007	**0.021**^**c**^	0.014	0.004	0.003	0.012	0.007
	(0.004)	(0.004)	(0.010)	(0.009)	(0.004)	(0.003)	(0.008)	(0.008)
Physical Symptoms Each Year		**0.231**^**a**^		**0.231**^**a**^		**0.164**^**a**^		**0.163**^**a**^
		(0.023)		(0.023)		(0.020)		(0.020)
Daily Pollutant level × Physical Symptoms		**0.003**^**a**^		**0.009**^**b**^		**0.002**^**b**^		**0.006**^**b**^
		(0.001)		(0.003)		(0.001)		(0.002)
Constant	**1.444**^**a**^	**1.515**^**a**^	**1.563**^**a**^	**1.608**^**a**^	**1.340**^**a**^	**1.388**^**a**^	**1.390**^**a**^	**1.421**^**a**^
	(0.127)	(0.115)	(0.070)	(0.064)	(0.108)	(0.101)	(0.059)	(0.055)

Standard errors in parentheses. ^a^*p* < 0.001, ^b^*p* < 0.01, ^c^*p* < 0.05. Significant associations are also bolded.

**Figure 1:**
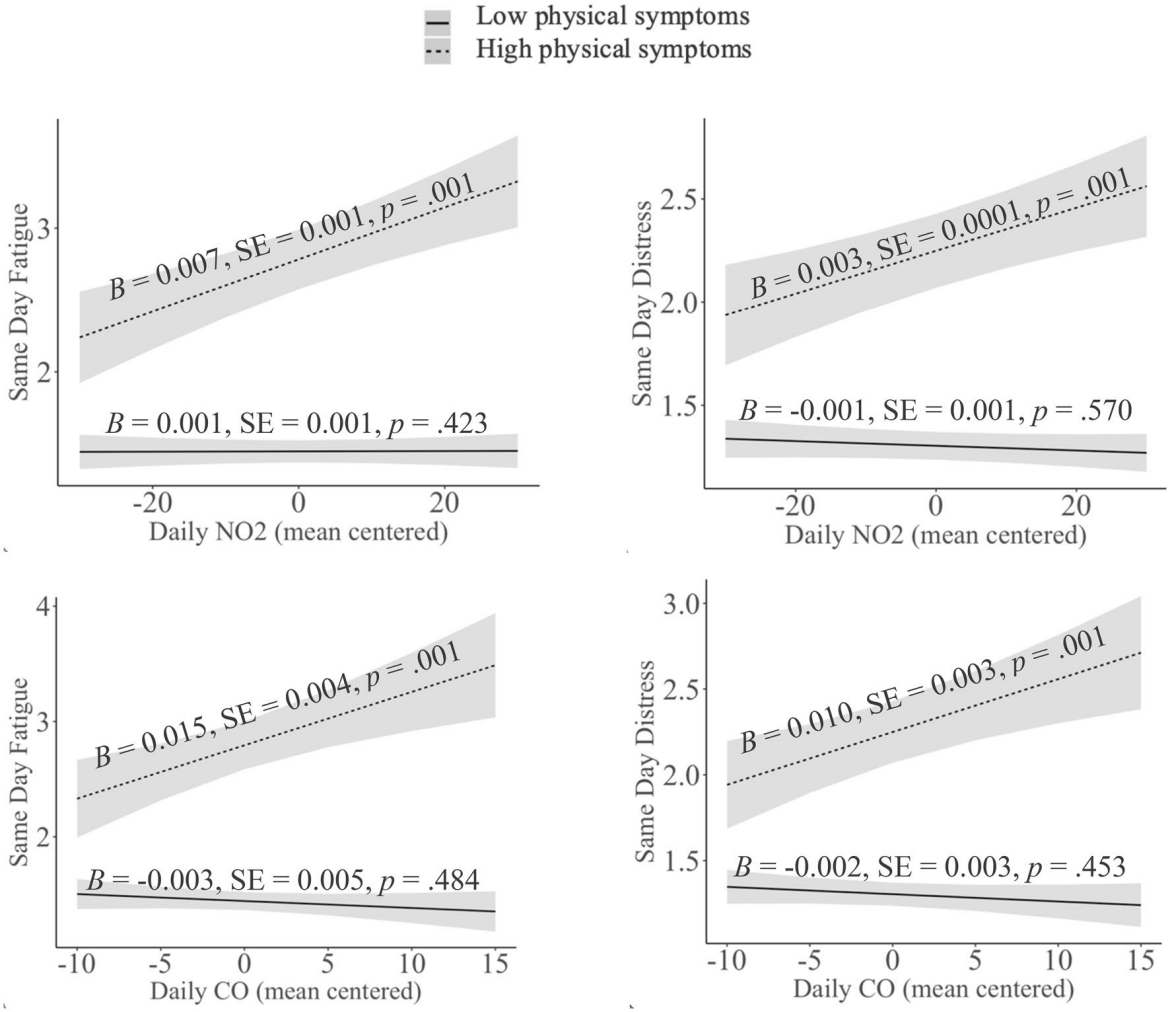
Daily NO_2_ and CO levels are positively related to daily feelings of fatigue and emotional distress among adolescents who report greater ongoing physical symptoms for the year (+ 1 *SD* above the mean level of physical symptoms) but not among adolescents who report fewer ongoing physical symptoms for the year (− 1 *SD* below the mean level of physical symptoms).

As shown in Table 1 Model 2, daily NO_2_ and CO each interacted with yearly physical symptoms to predict daily fatigue and emotional distress. Specifically, as shown in Fig. 1 (Panels A through D), higher levels of NO_2_ and CO in the air were related to greater fatigue and emotional distress the same day only among adolescents who experienced higher levels of ongoing physical symptoms that year (e.g., headache, backache), and not among adolescents who experienced lower levels of physical symptoms that year.

We conducted sensitivity analyses by additionally controlling for daily temperature (in Fahrenheit) and relative humidity, as reported by the EPA^33^. Specifically, we ran the same primary regression models as described above, but additionally included these two covariates. As displayed in the Supplementary Materials (Table S4), all findings remained significant (*p* < 0.01), except for the direct daily association between NO_2_ and fatigue which became marginally significant (*p* = 0.051) but continued to be qualified by the significant interaction.

### Same-day multilevel mediation analyses

We tested whether the same-day associations between each air pollutant and emotional distress were mediated via a significant pathway through same-day levels of fatigue. As shown in Table 2 Panel A, the indirect and total effects of NO_2_ and CO on daily emotional distress via daily fatigue were significant (*p*s < 0.01). Specifically, adolescents felt higher levels of fatigue on days there was more NO_2_ and CO in the air, and felt higher levels of daily emotional distress on days they felt more fatigue, which partially explained the same-day association between NO_2_ and CO levels and emotional distress. This finding suggests that daily NO_2_ and CO levels are associated with greater daily emotional distress each day in part because adolescents feel more fatigued that day.

**Table 2:** Multilevel mediation results testing whether NO_2_ and CO are related to emotional distress the same day (Panel A) and the next day (Panel B) via fatigue the same day, controlling for distress the same day.

	Daily NO_2_ → Daily Fatigue → Same Day Emotional Distress			Daily CO → Daily Fatigue → Same Day Emotional Distress		
	*B*	*SE*	*p*	*B*	*SE*	*p*
**Panel A: Same Day Mediation Models**						
Total Effect	0.362	0.007	0.001	0.263	0.007	0.001
Total Indirect Effect Via Daily Fatigue	0.002	0.001	0.001	0.003	0.001	0.018
Direct Effect	0.002	0.001	0.011	0.005	0.002	0.025

	Daily NO_2_ → Daily Fatigue → Next Day Emotional Distress			Daily CO → Daily Fatigue → Next Day Emotional Distress		
	*B*	*SE*	*p*	*SE*	*B*	*p*
**Panel B: Next Day Mediation Models**						
Total Effect	0.058	0.008	0.001	0.058	0.008	0.001
Total Indirect Effect Via Daily Fatigue	0.001	0.000	0.001	0.001	0.000	0.024
Direct Effect	0.000	0.000	0.899	0.001	0.002	0.602

### Next-day multi-level regressions

Next, we tested whether air pollutants were related to fatigue and distress the next day, over and above levels of fatigue and distress the same day. As shown in the Supplementary Materials (Table S5), NO_2_ the same day (measured on day X) positively predicted levels of fatigue the next day (on day X + 1), over and above levels of fatigue the same day (i.e., day X; *B* = 0.010, *SE* = 0.004, *p* = 0.024). This direct association was not qualified by any significant interactions with physical symptoms (*p* > 0.05). In addition, the interaction between daily CO and yearly physical symptoms significantly predicted levels of distress the next day over and above levels of distress the same day (*B* = 0.005, *SE* = 0.002, *p* = 0.022). Probing this interaction (Fig. S1) revealed that daily CO was linked to marginally higher levels of distress the next day among adolescents with high physical symptoms (*B* = 0.006, *SE* = 0.003, *p* = 0.061), but not among adolescents with low levels of physical symptoms (*B* = -0.004, *SE* = 0.003, *p* = 0.210).

### Next-day multilevel mediation analyses

We also tested the mediation model with days lagged across time. Specifically, we conducted the same multi-level mediation analyses as above, but tested whether levels of each air pollutant on day X were related to distress the next day (i.e., day X + 1) via an indirect path through daily fatigue the same day as the air pollutant (i.e., day X), over and above levels of distress the same day (i.e., day X). As shown in Table 2 Panel B, we found that the indirect pathways and total effects for NO_2_ and CO remained significant. Specifically, NO_2_ and CO were related to greater fatigue the same day, which in turn partially explained the association with increased distress the next day.

### Additional analyses for particulate air pollution

As supplementary analyses, we also investigated two types of particulate matter air pollution, PM_2.5_ and PM_10_. We assessed whether PM_2.5_ and PM_10_ were directly or interactively (with yearly physical symptoms) related on a daily level to adolescents’ fatigue and emotional distress. Specifically, we fitted the same primary regression models as outlined above, but modeled daily and average levels of PM_2.5_ and PM_10_ as predictors. As shown in the supplementary materials (Table S6), PM_2.5_ and PM_10_ were not directly or interactively associated with fatigue or emotional distress on a daily level (*p* > 0.10).

## Discussion

This within-subjects, daily-level study investigated whether adolescents’ experiences of fatigue and emotional distress fluctuated within and across days with air quality. We found that adolescents with ongoing physical complaints reported greater fatigue and emotional distress on days the air contained higher levels of NO_2_ and CO in LA county. They also felt greater emotional distress (but not fatigue) one day after levels of CO were higher. The same-day and day-lagged associations between air pollution (i.e., NO_2_ and CO) and emotional challenges were partially explained via an indirect pathway through increased fatigue the same day. In addition, regardless of their physical symptoms, all adolescents on average experienced more fatigue one day after the air contained greater NO_2_. These robust, within-person associations are consistent with the hypothesis that small increases NO_2_ and CO air pollution are daily-level risk-factors for youths’ experiences of fatigue and tiredness, which in turn may contribute to emotional problems within and across days.

### Daily NO_2_ and CO linked to fatigue and emotional distress

Air pollution was related positively to fatigue and distress the same day only among adolescents who experienced relatively higher levels of ongoing physical symptoms (e.g., headaches, stomachaches, backpain) that year, and not among adolescents who experienced lower levels of physical symptoms. These findings are consistent with prior studies that used between-subject analyses, in which children and adults with ongoing illnesses suffered more serious symptoms as a result of air pollution over time^35^. Our findings extend this prior work by suggesting that adolescents with greater physical symptoms may be more sensitive to the effects of air pollution that occur the same day. Adolescents with greater physical symptoms may be more sensitive to air pollution because they are experiencing compromised immune functioning, heightened inflammation, or inadequate sleep or rest, all of which can contribute to fatigue and distress the same day.

Specifically, we found that among adolescents with ongoing health concerns, ambient levels of NO_2_ and CO (but not SO_2_, O_3_, PM_2.5_, and PM_10_) were associated with higher levels of fatigue and emotional distress the same day. NO_2_ and CO originate primarily from the same source—vehicles^20^—which are also the main source pollution in LA County (LA County Department of Public Health, 2022). LA county where traffic density is high, motor vehicle-based NO_2_ and CO emissions contribute to more than 50% of all pollution (LA County Department of Public Health, 2022). In the context of high levels of vehicle-based NO_2_ and CO emissions, NO_2_ and CO may be particularly impactful in adolescents’ physical energy and emotional health, particularly for those who are experiencing poorer health overall. In contrast, SO_2_, PM_2.5_, and PM_10_ are more commonly emitted from stationary sources such as electric, manufacturing, and processing facilities^20^, which are relatively lower contributors to overall air pollution in LA County. O_3_ is less strongly correlated with other pollutants^20^ and may not be as directly relevant for adolescents’ emotional health. This work extends prior evidence that even small exposures to NO_2_ and CO can impact human health^33^, even when NO_2_ and CO levels were still relatively healthy or moderate.

Our findings that only NO_2_ and CO (and not O_3_, SO_2_, PM_2.5_ and PM_10_) were associated on a daily level with adolescents’ physical and emotional health is also consistent with prior research that differentiates the effects of different pollutants on human health. A meta-analysis of 22 studies from 10 countries worldwide revealed that short-term exposure (< 30 days) to NO_2_, but not other air pollutants, was significantly associated with depressive symptoms among adults and children^10^. This cross-sectional meta-analysis is supported by a few daily level studies from Asia, which have found that NO_2_ is uniquely linked to physical and emotional health on a daily level. In China, adults and children suffered from more respiratory health problems on days that NO_2_ levels were higher, but not on days that other pollutants such as SO_2_ and PM_10_ were higher^34^. In addition, hospital admissions for schizophrenia increased on days that levels of NO_2_ were higher, but not on days that CO or PM_10_ were higher^21^.

### The mechanisms linking pollution to emotional distress

We also found that the daily associations between NO_2_ and CO and adolescents’ emotional distress were mediated significantly by an indirect pathway through daily fatigue. Specifically, adolescents felt more fatigued on days that ambient NO_2_ and CO levels were higher, and felt higher levels of emotional distress on days they experienced more fatigue, which partially explained why NO_2_ and CO levels were linked to greater emotional distress the same day and also the next day. These findings shed light on the mechanisms linking air pollution to mood. In particular, NO_2_ and CO may relate to emotional health partially via pathways of increasing feelings of fatigue, exhaustion and being worn-out. Incremental exposures to NO_2_ impair the body’s ability to fight off pulmonary infections and can contribute to tiredness, difficulty breathing, and inflammation^35^. On a physiological level, pollution-associated fatigue likely occurs in part because pollution increases inflammation and adversely impacts physiological functioning^24^. Exposure to ambient air pollution is linked positively with inflammation across the lifespan, pollution increases levels of short-term and long-term inflammatory markers^36^. In turn, inflammation contributes to adolescents’ feelings of tiredness and physical pain by multiple physiological pathways ranging from neuroendocrine response changes to brain changes, which in turn hinders their capacity to regulate and maintain emotional wellbeing^24^. Beyond inflammation, other potential biological mechanisms linking air pollution to emotional health include altered oxidative stress, blood coagulation, autonomic dysfunction^36^ and gene regulation^37^, all of which tax physiological functioning, contribute to fatigue, and decrease adolescents’ capacity to cope emotionally with environmental stressors. Notably, our findings are consistent with the hypothesis that these underlying physiological processes likely occur both within- and across days. Indeed, regardless of physical symptoms, adolescents on average reported greater fatigue the day after NO_2_ levels were higher (in addition to the same day).

### Implications

Our findings have implications for research from environmental, epidemiological, and physical and mental health disciplines. For decades, researchers from epidemiology, psychology, and health sectors have recognized that air pollution is a risk-factor for long term physical and emotional health^5,6,8,15^. Our study contributes to this interdisciplinary body of work literature in three primary ways. First, we offer evidence that increases in air pollution are linked on a daily level to adolescents’ fatigue and mood, in particular among relatively less healthy adolescents, which builds on prior research which focused on between-subject analyses^5–9^ and within-subjects analyses among adults in Asia^18,21,22,34^. Our findings offer greater temporal specificity by revealing that there are same-day and next-day associations between air quality and youths’ fatigue and emotional health in the US context. Second, our findings identify NO_2_ and CO as two air pollutants that might be particularly salient for adolescents’ fatigue and mood on a daily level (compared to other pollutants), consistent with prior research that measured associations across days^10^ and/or focused on adults^21,22,34^. These findings inform policy efforts designed to curtail specific pollutants and promote human health. Third, our interdisciplinary study illustrates how daily self-reported data from psychological studies can be combined with publicly available environmental data to shed greater light on how the physical environment impacts youths’ health and development.

### Limitations and directions for future research

Our study has several limitations and offers suggestions for future research. First, our small effect sizes suggest that other factors (beyond NO_2_ and CO) affect adolescents’ daily levels of fatigue and distress. For instance, we did not measure inflammatory markers or other biological factors (e.g., cortisol) which may mediate the daily links between pollution and adolescents’ experiences. Future work could repeatedly measure inflammatory markers via finger-pricks every day^38^. Second, the average air quality in our sample was relatively healthy across days; only NO_2_ and O_3_ approached moderately unhealthy and unhealthy levels. Additional associations (e.g., with SO_2_) may emerge when air quality is worse. Relatedly, our data were collected from late 2009 to 2011; future studies should replicate our findings in recent years as the observed associations may change over time. Fourth, while our study focused on the adolescent period, future research should examine developmental differences. In one prior study, air pollution was linked to more physical health problems among younger children compared to adolescents and adults^39^. Similar age differences may be revealed for fatigue and mood. Fifth, we linked county-level air pollution to individuals, and did not use individual-level air pollution tracked by their location. Future research could use geospatial tracking devices and code different exposure levels based of that specific locations and/or how much time adolescents spend outside. Finally, our study focused on Mexican–American adolescents in LA. Future studies should replicate how findings to clarify whether they are generalizable to other communities in the US and worldwide. Future studies could also investigate potential interactive effects between multiple pollutants; for example, it is possible that the health effects of NO_2_ and CO are compounded when in combination with one another.

## Methods

### Participants

Participants were from a sample of 428 Mexican–American adolescents in Los Angeles (LA) County, California. Of these adolescents, 6 did not have daily-report data with usable, valid dates that could be linked to EPA air quality data, resulting in a total analytical sample of 422 adolescents (50% female; *Mage* = 15.98 ± 1.56 years; *Range* 14—20 years). Participants were recruited from two public high schools in LA County. The student body of both schools was majority Latin American (62% and 94%) from lower- to lower-middle class families. Approximately 29% of primary caregivers completed eighth-grade education, 26% completed high school, 22% completed postsecondary education or more (parental education data was missing for the remainder). In both schools, more than 70% of students qualified for free- and reduced-priced meals^40^.

### Procedures

This study uses three sources of data: (1) daily diaries that were linked by date to (2) objective daily air quality data collected by the EPA, and (3) yearly self-report questionnaires. The daily-report data were collected for 28 days over the course of two years in two waves (14 days in each yearly wave). All data were collected from November 2009–December 2011. The study days were in March–May (48.70% of participant days), June–August (23.28%) and November–February (28.02%) over the course of the two years. Study time varied from individual to individual, depending on when participants enrolled. By chance, no data were collected in September or October.

Adolescents who elected to participate in the study were provided with 14 diary checklists for two-week periods twice over the course of two years, such that they completed 28 diaries over the course of two years. There were 9696 daily observations. The daily diaries took 5 to 10 min to complete each day in English or Spanish. Adolescents sealed each completed daily dairy each night and stamped the seal with an electronic time stamper that imprinted the current date and time that could not be altered. Adolescents completed 95% of their daily diaries. Research assistants collected all the daily diaries at the end of the two-week study period. In addition to the daily diaries, adolescents completed a longer questionnaire that inquired about their demographics, traits and experiences each of the two years. Adolescents provided assent and parents provided informed written consent, and were all compensated for their time. All procedures were approved by the University of California at LA Institutional Review Board. All methods were performed in accordance with the relevant guidelines and regulations.

### Measures

#### Daily air quality

Using AQIs for each pollutant, we focused on daily measurements of NO_2_, CO, O_3_, and SO_2_ which are the four major criteria gasses monitored daily via the EPA. Levels of these pollutants are reported on a daily level for multiple data collection locations within LA County (N = 14–18 depending on the day). First, we downloaded daily data from the EPA website which contained multiple measurements per county from different data location cites within each county^33^. Second, we restricted the values only to LA County, and averaged across data collection locations within LA County. This yielded daily, county-level values of NO_2_, CO, O_3_, and SO_2_. Averaging across stations within a county is an approach consistent with prior research^17^. For each pollutant, we calculated two variables: daily level and person-level. The daily level variable was person-mean-centered and reflected the relative level of pollutant exposure the adolescent experienced each day relative to their average levels of exposure. We calculated person-mean-centered values by subtracting each adolescent’s mean value of pollutant exposure from their daily value of exposure to that pollutant. The person-level variable reflected each adolescents’ average level of exposure across all their study days. We calculated person-mean values by averaging across all 28 days of the study period for each adolescent. For each pollutant, both the mean-centered variable and the person-average variable were included as simultaneous predictors in our regression models to disaggregate within-subjects vs between-subjects effects, as described further in the analysis plan. The inter-item correlations (ICCs) reflecting the correlation of air pollution within each adolescent across days of study were 0.39 for NO_2_, 0.62 for CO, 0.07 for SO_2_, and 0.53 for O_3_. The ICCs reflecting the correlation among average levels of air pollution between adolescents across days of study was 0.95 for NO_2_, 0.98 for CO, 0.69 for SO_2_, and 0.97 for O_3_. As described in further detail in the results, for CO and SO2, all daily observations of fell within the “healthy” category, so no days exceeded health recommendations^33^. For NO_2_ and O_3_, 1.90% and 26.93% of observations fell within the “moderately unhealthy” category respectively^33^.

#### Daily fatigue

Each evening during each 2-week period, adolescents’ daily fatigue was assessed with items from the Profile of Mood States^41^, a widely used measure in previous daily diary studies of stress and psychological well-being^42–44^. Adolescents used a 5-point scale ranging from 1 (not at all) to 5 (extremely) to indicate the extent to which they experienced different feelings each day. A measure of d*aily fatigue* was calculated from three items (fatigue, exhausted, and worn-out; alpha = 0.76). This was a continuous, daily-level variable. The ICC reflecting the correlation of daily fatigue within adolescents across days of study was 0.35. The ICC reflecting the correlation between average levels of fatigue between adolescents across days of study was 0.94.

#### Daily emotional distress

Each evening during each 2-week period, adolescents’ daily emotional distress was also assessed with items from the Profile of Mood States^41^. Adolescents used a 5-point scale ranging from 1 (not at all) to 5 (extremely). A measure of daily fatigue was calculated from three items (fatigue, exhausted, and worn-out; alpha = 0.76). The ICC reflecting the correlation of daily fatigue within adolescents across days of study was 0.35 and between adolescents was 0.94. A measure of emotional distress was calculated from nine items (sad, hopeless, discouraged, on edge, unable to concentrate, uneasy, nervous, stressed, worried). The ICC reflecting the correlation of daily distress within adolescents across days of study was 0.55 and between adolescents was 0.96.

#### Yearly physical symptoms

Adolescents also self-reported on their ongoing *Physical Symptoms* one time each year at a separate time point from the daily diary measures. This measure was frequency of physical complaints. Across the past two weeks. Specifically, adolescents were prompted “Please rate how many times in the past two weeks you experienced each of the following physical complaints, using the rating scale below”. There were 12 items that adolescents rated on a four-point Likert-type scale with responses indicating “Not at all” , “Once or twice”, “A few times”, to “Almost every day”. The items were “Headaches”, “Very tired for no reason”, “Dizziness”, “Stomachaches or pain”, “Upset stomach/ nausea”, “Sore throat/ coughs”, “Low energy”, “Poor appetite”, “Sleep problems”, “Other aches and pains”, “Cold sweats”, “Trouble catching breath”. Accordingly, each adolescent had two values for *Physical Symptoms:* one for the first year (alpha = 0.78), and one for the second year (alpha = 0.87). The ICC reflecting the correlation of yearly physical symptoms within adolescents across years of study was 0.73 and between adolescents was 0.98.

#### Covariates

We only included daily-level covariates in the model, because our analyses were within-subjects. Between-subject covariates were not necessary because these were within-subject models that controlled for between-subject effects^16,45^. In primary analysis, we controlled for whether or not it was a *school day* (1 = school day, 0 = not a school day) because this could impact the adolescents’ locations and time outside. In sensitivity analyses, we additionally controlled for the temperature (in Fahrenheit) and level of relative humidity each day (both continuous measures). These were collected by the EPA for each day in LA County and accessed via the same source at pollution variables^33^.

### Analyses

For our primary analysis, we fitted linear mixed effect regression models that nested days (Level 1) within years (Level 2) within participants (Level 3). We person-centered all Level 1 pollutants and included on the intercept person-mean values for each pollutant. This approach is crucial for isolating within-subject vs between subject effects^45^. The outcomes were not standardized. Model 1 assessed each air pollutant as person-mean-centered Level 1 predictors of adolescents’ fatigue and emotional distress the same day in separate models, controlling for day of the week and person-average levels of each air pollutant. We tested each pollutant in separate models. Model 2 additionally included cross-level interaction terms between each daily Level 1 air pollutant and the Level 2 moderator (i.e., yearly levels of physical symptoms). We then tested the same models, but with day-lagged effects. Specially, each air pollutant was fitted Level 1 in separate models as predictors of adolescents’ fatigue and emotional distress the next day, controlling for levels of fatigue and emotional distress the same day. We probed significant interactions by plotting the association between air pollution and fatigue or emotional distress above and below 1*SD* for the values of the moderator (i.e., physical symptoms).

For mediation analysis, we tested multi-level mediation models again with days (Level 1) nested within years (Level 2) within participants (Level 3). Specifically, first we tested whether the daily associations between each air pollutant and daily emotional distress were mediated via a significant pathway through same daily levels of fatigue. Next, we tested one-day time-lagged models such that each daily air pollutant predicted next-day daily emotional distress, and whether this was mediated via a significant pathway through same daily levels of fatigue. We evaluated these mediation models by examining the significance level and coefficients of the indirect and total effects. Missing data ranged from 0.30%—1.82% and was handled using Full Information Maximum Likelihood (FIML). All analyses were conducted in Stata Version 17. See Supplementary Materials for more details.

## Supplementary Information

Supplementary Information.

## Data Availability

All data and syntax are available upon request from the corresponding author.

## References

[1] Hoffmann B, Boogaard H, de Nazelle A, Andersen ZJ, Abramson M, Brauer M, Brunekreef B, Forastiere F, Huang W, Kan H, Kaufman JD, Katsouyanni K, Krzyzanowski M, Kuenzli N, Laden F, Nieuwenhuijsen M, Mustapha A, Powell P, Rice M, Thurston G (2021). WHO air quality guidelines 2021–aiming for healthier air for all: A joint statement by medical, public health, scientific societies and patient representative organisations. Int. J. Public Health.

[2] Huang M, Chen J, Yang Y, Yuan H, Huang Z, Lu Y (2021). Effects of ambient air pollution on blood pressure among children and adolescents: A systematic review and meta-analysis. J. Am. Heart Assoc..

[3] Lopuszanska U, Samardakiewicz M (2020). The relationship between air pollution and cognitive functions in children and adolescents: A systematic review. Cogn. Behav. Neurol..

[4] Stenson C, Wheeler AJ, Carver A, Donaire-Gonzalez D, Alvarado-Molina M, Nieuwenhuijsen M, Tham R (2021). The impact of traffic-related air pollution on child and adolescent academic performance: A systematic review. Environ. Int..

[5] Klompmaker JO, Hoek G, Bloemsma LD, Wijga AH, van den Brink C, Brunekreef B, Lebret E, Gehring U, Janssen NAH (2019). Associations of combined exposures to surrounding green, air pollution and traffic noise on mental health. Environ. Int..

[6] Latham RM, Kieling C, Arseneault L, Botter-Maio Rocha T, Beddows A, Beevers SD, Danese A, De Oliveira K, Kohrt BA, Moffitt TE, Mondelli V, Newbury JB, Reuben A, Fisher HL (2021). Childhood exposure to ambient air pollution and predicting individual risk of depression onset in UK adolescents. J. Psychiatr. Res..

[7] Lim J, Kweon K, Kim H-W, Cho SW, Park J, Sim CS (2018). Negative impact of noise and noise sensitivity on mental health in childhood. Noise Health.

[8] Ni N, Chi X, Liu W, Cui X (2021). Air pollution and adolescent development: Evidence from a 3-year longitudinal study in China. Children.

[9] Roberts S, Arseneault L, Barratt B, Beevers S, Danese A, Odgers CL, Moffitt TE, Reuben A, Kelly FJ, Fisher HL (2019). Exploration of NO_2_ and PM_2.5_ air pollution and mental health problems using high-resolution data in London-based children from a UK longitudinal cohort study. Psychiatry Res..

[10] Fan S-J, Heinrich J, Bloom MS, Zhao T-Y, Shi T-X, Feng W-R, Sun Y, Shen J-C, Yang Z-C, Yang B-Y, Dong G-H (2020). Ambient air pollution and depression: A systematic review with meta-analysis up to 2019. Sci. Total Environ..

[11] Hand JL, Prenni AJ, Copeland S, Schichtel BA, Malm WC (2020). Thirty years of the Clean Air Act Amendments: Impacts on haze in remote regions of the United States (1990–2018). Atmos. Environ..

[12] Clay, K., & Muller, N. Z. *Recent Increases in Air Pollution: Evidence and Implications for Mortality* (Working Paper No. 26381) (National Bureau of Economic Research, 2019). 10.3386/w26381.

[13] Dahl RE, Allen NB, Wilbrecht L, Suleiman AB (2018). Importance of investing in adolescence from a developmental science perspective. Nature.

[14] Mueller MAE, Flouri E, Kokosi T (2019). The role of the physical environment in adolescent mental health. Health Place.

[15] Lin W-H, Pan W-C, Yi C-C (2019). “Happiness in the air?” the effects of air pollution on adolescent happiness. BMC Public Health.

[16] Hoffman L, Stawski RS (2009). Persons as contexts: Evaluating between-person and within-person effects in longitudinal analysis. Res. Hum. Dev..

[17] Liang L, Cai Y, Barratt B, Lyu B, Chan Q, Hansell AL, Xie W, Zhang D, Kelly FJ, Tong Z (2019). Associations between daily air quality and hospitalisations for acute exacerbation of chronic obstructive pulmonary disease in Beijing, 2013–17: An ecological analysis. Lancet Planet. Health.

[18] Pande JN, Bhatta N, Biswas D, Pandey RM, Ahluwalia G, Siddaramaiah NH, Khilnani GC (2002). Outdoor air pollution and emergency room visits at a hospital in Delhi. Indian J. Chest Dis. Allied Sci..

[19] Yadav R, Nagori A, Mukherjee A, Singh V, Lodha R, Kabra SK, Yadav G, Saini JK, Singhal KK, Jat KR, Madan K, George MP, Mani K, Mrigpuri P, Kumar R, Guleria R, Pandey RM, Sarin R, Dhaliwal RS (2021). Effects of ambient air pollution on emergency room visits of children for acute respiratory symptoms in Delhi, India. Environ. Sci. Pollut. Res..

[20] Stieb DM, Burnett RT, Smith-Doiron M, Brion O, Shin HH, Economou V (2008). A new multipollutant, no-threshold air quality health index based on short-term associations observed in daily time-series analyses. J. Air Waste Manag. Assoc..

[21] Bai L, Zhang X, Zhang Y, Cheng Q, Duan J, Gao J, Xu Z, Zhang H, Wang S, Su H (2019). Ambient concentrations of NO_2_ and hospital admissions for schizophrenia. Occup. Environ. Med..

[22] Gu X, Guo T, Si Y, Wang J, Zhang W, Deng F, Chen L, Wei C, Lin S, Guo X, Wu S (2020). Association between ambient air pollution and daily hospital admissions for depression in 75 Chinese cities. Am. J. Psychiatry.

[23] Brockmeyer S, D’Angiulli A (2016). How air pollution alters brain development: The role of neuroinflammation. Transl. Neurosci..

[24] Rosenblat JD, Cha DS, Mansur RB, McIntyre RS (2014). Inflamed moods: A review of the interactions between inflammation and mood disorders. Prog. Neuropsychopharmacol. Biol. Psychiatry.

[25] Irwin MR (2019). Sleep and inflammation: Partners in sickness and in health. Nat. Rev. Immunol..

[26] Becker SP, Langberg JM, Byars KC (2015). Advancing a biopsychosocial and contextual model of sleep in adolescence: A review and introduction to the special issue. J. Youth Adolesc..

[27] Lob E, Kirschbaum C, Steptoe A (2020). Persistent depressive symptoms, HPA-axis hyperactivity, and inflammation: The role of cognitive-affective and somatic symptoms. Mol. Psychiatry.

[28] Steptoe A, Kivimäki M, Batty GD, Steptoe A, Kawachi I (2017). Depression and negative emotions. The Routledge International Handbook of Psychosocial Epidemiology.

[29] Ames ME, Leadbeater BJ, MacDonald SWS (2018). Health behavior changes in adolescence and young adulthood: Implications for cardiometabolic risk. Health Psychol..

[30] Kozlowska K, Scher S, Helgeland H, Kozlowska K, Scher S, Helgeland H (2020). The Immune-Inflammatory System and Functional Somatic Symptoms. Functional Somatic Symptoms in Children and Adolescents: A Stress-System Approach to Assessment and Treatment.

[31] Burbank AJ, Peden DB (2018). Assessing the impact of air pollution on childhood asthma morbidity: How, when and what to do. Curr. Opin. Allergy Clin. Immunol..

[32] Tiotiu AI, Novakova P, Nedeva D, Chong-Neto HJ, Novakova S, Steiropoulos P, Kowal K (2020). Impact of air pollution on asthma outcomes. Int. J. Environ. Res. Public Health.

[33] US Environmental Protection Agency. *AirData Website File Download page* [Data & Tools]. https://aqs.epa.gov/aqsweb/airdata/download_files.html#Daily (2022).

[34] Zhao W, Cheng J, Li D, Duan Y, Wei H, Ji R, Wang W (2013). Urban ambient air quality investigation and health risk assessment during haze and non-haze periods in Shanghai, China. Atmos. Pollut. Res..

[35] Ćurić M, Zafirovski O, Spiridonov V, Ćurić M, Zafirovski O, Spiridonov V (2022). Air quality and health. Essentials of Medical Meteorology.

[36] Chuang K-J, Chan C-C, Su T-C, Lee C-T, Tang C-S (2007). The effect of urban air pollution on inflammation, oxidative stress, coagulation, and autonomic dysfunction in young adults. Am. J. Respir. Crit. Care Med..

[37] Reuben A, Sugden K, Arseneault L, Corcoran DL, Danese A, Fisher HL, Moffitt TE, Newbury JB, Odgers C, Prinz J, Rasmussen LJH, Williams B, Mill J, Caspi A (2020). Association of neighborhood disadvantage in childhood with DNA methylation in young adulthood. JAMA Netw. Open.

[38] McDade TW, Miller A, Tran TT, Borders AEB, Miller G (2021). A highly sensitive multiplex immunoassay for inflammatory cytokines in dried blood spots. Am. J. Hum. Biol..

[39] Veremchuk LV, Tsarouhas K, Vitkina TI, Mineeva EE, Gvozdenko TA, Antonyuk MV, Rakitskii VN, Sidletskaya KA, Tsatsakis AM, Golokhvast KS (2018). Impact evaluation of environmental factors on respiratory function of asthma patients living in urban territory. Environ. Pollut. (Barking, Essex: 1987).

[40] California Department of Education. *Student Poverty*. https://www.cde.ca.gov/ds/ad/afilessp1112.asp (2011).

[41] Lorr M, McNair DM, Droppleman LF (1971). Manual: Profile of Mood States.

[42] Bolger N, Zuckerman A (1995). A framework for studying personality in the stress process. J. Pers. Soc. Psychol..

[43] Fuligni AJ, Pedersen S (2002). Family obligation and the transition to young adulthood. Dev. Psychol..

[44] Telzer EH, Fuligni AJ (2009). Daily family assistance and the psychological well-being of adolescents from Latin American, Asian, and European backgrounds. Dev. Psychol..

[45] Curran PJ, Bauer DJ (2011). The disaggregation of within-person and between-person effects in longitudinal models of change. Annu. Rev. Psychol..

