# Supplementary material for: A 28-day, 2-year study reveals that adolescents are more fatigued and distressed on days with greater NO2 and CO air pollution
Source: Sci Rep. 2022 Oct 11;12:17015. doi: 10.1038/s41598-022-20602-z (PMC9553977; doi:10.1038/s41598-022-20602-z)
Supplement: Supplementary file 1 — Supplementary Information. [file 41598_2022_20602_MOESM1_ESM.docx]

Online Supplement:

**A 28-day, two-year study reveals that adolescents are more fatigued and distressed on days with greater NO_2_ and CO air pollution**

**Methods**

**Adolescent daily diary (relevant items).**

**DATE: ________________ Adolescent Daily Diary Checklist**

**Day 1**

**TIME:  IS THIS A SCHOOL DAY? YES NO ID____________________**

**The following is a list of feelings or experiences. How much did you experience them today?**

*Not at all A little Moderately Quite a bit Extremely*

On edge 1 2 3 4 5

Joyful 1 2 3 4 5

Exhausted 1 2 3 4 5

Sad 1 2 3 4 5

Unable to concentrate 1 2 3 4 5

Worn-out 1 2 3 4 5

Happy 1 2 3 4 5

Uneasy 1 2 3 4 5

Fatigued 1 2 3 4 5

Hopeless 1 2 3 4 5

Nervous 1 2 3 4 5

Discouraged 1 2 3 4 5

Calm 1 2 3 4 5

Headache 1 2 3 4 5

Back, joint, or muscle pain 1 2 3 4 5

Trouble sleeping 1 2 3 4 5

**Adolescent trait-level questionnaire (relevant items).**

Please rate how many times in the past two weeks you experienced each of the following physical complaints, using the rating scale below. Please response on a four-point Likert-type scale with responses indicating “Not at all” , “Once or twice”, “A few times”, to “Almost every day”.

1. “Headaches”
2. “Very tired for no reason”
3. “Dizziness”
4. “Stomachaches or pain”
5. “Upset stomach/ nausea”
6. “Sore throat/ coughs”
7. “Low energy”
8. “Poor appetite”
9. “Sleep problems”
10. “Other aches and pains”
11. “Cold sweats”
12. “Trouble catching breath”.

**Air pollution details.**

We used AQIs in our analyses, rather than raw concentrations of pollution levels. The AQI is a standardized unit that allows for consistency and comparisons across specific pollutants. We used AQIs because this is the way that the EPA reports daily pollutant data and recommends analyzing daily pollution, because it is a standardized unit. We gathered this information from the Technical Assistance document provided by the EPA, and confirmed via personal correspondence with Dr. Julia Black, a representative of the Air Quality Analysis Group on 2/2/2022 (U.S. Environmental Protection Agency, 2018).

An AQI value of 100 generally corresponds to an ambient air concentration that equals the level of the short-term national ambient air quality standard for protection of public health. AQI values at or below 100 are generally considered satisfactory. For instance, according to the AirNow.Gov calculator (https://www.airnow.gov/aqi/aqi-calculator/): an AQI of 100 for SO2 corresponds to a level of 75 parts per billion (averaged over one hour); an AQI of 100 for O3 corresponds to an O3 level of 70 parts per billion (averaged over 8 hours); an AQI of 100 for CO corresponds to a level of 9.4 parts per million (averaged over 8 hours); an AQI of 100 for NO_2_ corresponds to a NO_2_ level of 100 parts per million (averaged over one hour). Please see <https://www.who.int/publications/i/item/9789240034228> for information about how these values correspond to recommendations from the World Health Organization.

There were up to 18 EPA-regulated monitoring stations reporting daily pollution data during the study period. Not all stations reported values daily pollution data each day, but at least 14 reported each day.

**Equations for primary regression models**

Our first research question was whether daily levels of pollutant exposure predicted adolescents’ levels of fatigue and emotional distress the same day. We tested standardized values of adolescents’ fatigue, then emotional distress, as function of each person-centered pollutant exposure (NO_2_, SO_2_, CO and O_3_) the same day, controlling for person-average pollutant exposure across days (Model 1) and for whether it was a weekday. We used the equations below, in which *AQI_ijk_* denotes the *i* individual adolescent mean-centered pollutant value (i.e., NO_2_, SO_2_, O_3_, or CO), on day *j* and year *k.* *Distress* denotes the individual’s daily outcome (i.e., fatigue or distress).

Our equation for Model 1(testing direct associations) was:

Level 1 Equation:

Distress*_ijk_ = β_0jk_ + β_1jk_*AQI*_ijk_ + β_2jk_*Weekday*_ijk_ + r_ijk_*

Level 2 Equation:

*β_0jk_ = β_00k_+ u_0jk_*

*β_1jk_ = β_10k_*

*β_2jk_ = β_20k_*

Level 3 Equations:

*β_00k_ = γ_000_ +γ_001_*AQI*_k_ + u_00k_*

*β_10k_ = γ_100_*

*β_20k_ = γ_200_*

For Model 2, we additionally added a cross-level interaction term to this equation, to investigate whether the daily association between air pollution (Level 1) and adolescents’ outcomes (i.e., fatigue and distress on Level 1) differed for adolescents who experienced lower or higher levels of ongoing physical symptoms throughout the year (Level 2):

Level 1 Equation:

Distress*_ijk_ = β_0jk_ + β_1jk_*AQI*_ijk_ + β_2jk_*Weekday*_ijk_ + r_ijk_*

Level 2 Equation:

*β_0jk_ = β_00k_+ β_01k_*PhysicalSymptoms*_jk_ + u_0jk_*

*β_1jk_ = β_10k_+ β_11k_*PhysicalSymptoms*_jk_ + u_1jk_*

*β_2jk_ = β_20k_*

Level 3 Equations:

*β_00k_ = γ_000_ +γ_001_*AQI*_k_ + u_00k_*

*β_01k_ = γ_010_*

*β_10k_ = γ_100_*

*β_11k_ = γ_110_*

*β_20k_ = γ_200_*

**Results**

**Greater detail about descriptive information**

On average across days in the study, air quality was relatively good or moderate, as defined by pollutant-specific AQI thresholds determined by the EPA ^[34]^. Specifically, the levels of CO were low (*M* = 6.32, *SD* = 3.56, *Range =* 1.93 - 20.50), and all daily observations of CO fell within the “healthy” category defined as <50 AQI by the EPA for all pollutants ^[34]^. Similarly, levels of SO_2_ were low (*M* = 2.57, *SD* = 2.29, *Range =* 1.00 - 17.25) and all daily observations of SO_2_ fell within the “healthy” category. Levels of NO_2_ were relatively higher (*M* = 28.71, *SD* = 9.97, *Range =* 7.27 – 60.33), but most (98.10%) of the daily observations of NO_2_ still fell within the “healthy” category defined as <50 AQI, and 1.90% of the daily observations fell within the “moderately unhealthy” category (N = 184 daily observations), defined as 50 – 100 AQI. Levels of O_3_ were relatively higher (*M* = 42.60, *SD* = 16.89, *Range =* 8.23 – 114.13). Most (72.22%) of the daily observations of O_3_ were in the healthy category, whereas 26.93% (*N* = 2,608 daily observations), were in the moderately unhealthy category, and 0.85% (N = 82 daily observations) were in the unhealthy category defined as >100 AQI.

**Greater detail about the mediation results**

The indirect effect of NO_2_ on emotional distress via fatigue was significant (*B* = 0.002, *SE* = 0.001, p < 0.001). Specifically, adolescents felt higher levels of fatigue on days there was more NO_2_ in the air (*B* = 0.002, *SE* = 0.001, p = .011), and felt higher levels of emotional distress on days they felt more fatigue (*B* = 0.360, *SE* = 0.006, p < .001), which partially explained the association NO_2_ levels and emotional distress (*B* = 0.36, *SE* = 0.006, *p* < .001). In this mediation model, the direct association between NO_2_ levels and emotional distress was still significant (*B* = 0.002, SE = 0.001, p = .011). This suggests that providing NO_2_ levels are associated with greater emotional distress that day in part because adolescents feel greater fatigue on that day.

Similarly, the indirect effect of CO on emotional distress via fatigue was significant (*B* = 0.003, *SE* = 0.001, *p* < 0.018). Specifically, adolescents felt higher levels of fatigue on days there was more CO in the air (*B* = 0.005, *SE* = 0.002, *p* < 0.025), and felt higher levels of emotional distress on days they felt more fatigue (*B* = 0.360, *SE* = 0.007, *p* < .001), which partially explained the association CO levels and emotional distress (*B* = 0.363, *SE* = 0.007, *p* < .001). In this mediation model, the direct association between CO levels and emotional distress was still significant (*B* = 0.005, *SE* = 0.002, *p* < 0.025). This suggests that providing CO levels are associated with greater emotional distress that day in part because adolescents feel greater fatigue on that day.

*Table S1*

Descriptive statistics

|  | | | | *N* |
| --- | --- | --- | --- | --- |
| Observations | | | | 9,696 |
| Adolescents | | | | 422 |
| *Gender* | | | | |
| Female | | | | 211 |
| Male | | | | 211 |
| *Primary caregiver education level* | | | | |
| Completed 8^th^ grade | | | | *29%* |
| Completed high school | | | | *26%* |
| Completed postsecondary education or more | | | | *22%* |
| Missing | | | | *33%* |
| Students in schools who qualified for free- and reduced-price meals | | | | *70%* |
|  | *Mean* | *SD* | *Min* | *Max* |
| Adolescent age | 15.98 | 1.56 | 14.00 | 20.00 |
| NO_2_ | 28.71 | 6.60 | 18.10 | 46.19 |
| CO | 6.32 | 2.81 | 3.24 | 14.24 |
| O_3_ | 42.90 | 12.41 | 17.59 | 68.39 |
| SO_2_ | 2.59 | 0.80 | 1.00 | 7.33 |
| Weekday | 0.72 | 0.03 | 0.50 | 1.00 |
| Temperature | 60.92 | 5.43 | 50.83 | 73.76 |
| Humidity | 64.66 | 6.63 | 37.28 | 76.85 |
| Fatigue | 1.75 | 0.59 | 1.00 | 4.46 |
| Emotional Distress | 1.52 | 0.50 | 1.00 | 3.53 |
| Physical Symptoms | 1.59 | 0.41 | 1.00 | 3.09 |

*Note.* Temperature is in Fahrenheit. Humidity is in relative humidity, defined as the amount of water vapor present in air (i.e., a percentage of the amount needed for saturation at the same temperature). NO_2_ = Nitrogen Dioxide. CO = Carbon Monoxide. SO_2_= Sulfur Dioxide. O_3_ = Ground Ozone.

*Table S2*

Bivariate correlations between study constructs averaged within individual participants across days of the study.

|  | NO_2_ | CO | O_3_ | SO_2_ | Weekday | Temperature | Humidity | Fatigue | Distress |
| --- | --- | --- | --- | --- | --- | --- | --- | --- | --- |
| NO_2_ | 1 |  |  |  |  |  |  |  |  |
| CO | 0.91* | 1 |  |  |  |  |  |  |  |
| O_3_ | -0.80* | -0.90* | 1 |  |  |  |  |  |  |
| SO_2_ | 0.51* | 0.52* | -0.31* | 1 |  |  |  |  |  |
| Weekday | 0.08 | 0.04 | 0.00 | -0.05 | 1 |  |  |  |  |
| Temperature | -0.68* | -0.71* | 0.86* | -0.05 | 0.00 | 1 |  |  |  |
| Humidity | -0.71* | -0.55* | 0.43* | -0.28* | -0.19* | 0.37* | 1 |  |  |
| Fatigue | 0.09 | 0.09 | -0.077 | 0.06 | 0.00 | -0.05 | -0.06 | 1 |  |
| Emotional Distress | 0.06 | 0.06 | -0.051 | 0.03 | 0.00 | -0.04 | -0.02 | 0.79* | 1 |
| Physical Symptoms | 0.06 | 0.08 | -0.08 | 0.01 | -0.07 | -0.06 | -0.06 | 0.45* | 0.37* |

*Note.* * = *p*<0.001.

|  | Daily Fatigue | | | | Daily Emotional Distress | | | | |
| --- | --- | --- | --- | --- | --- | --- | --- | --- | --- |
|  | O_3_ -> Daily Fatigue | | SO_2_ -> Daily Fatigue | | O_3_ -> Daily Emotional Distress | | SO_2_ -> Daily Emotional Distress | | |
|  | Model 1 | Model 2 | Model 1 | Model 2 | Model 1 | Model 2 | Model 1 | Model 2 |  |
|  | *B* | *B* | *B* | *B* | *B* | *B* | *B* | *B* |  |
|  | *(SE)* | *(SE)* | *(SE)* | *(SE)* | *(SE)* | *(SE)* | *(SE)* | *(SE)* |  |
| Weekday | **0.080^a^** | **0.079^a^** | **0.075^a^** | **0.075^a^** | **0.076^a^** | **0.076^a^** | **0.079^a^** | **0.079^a^** |  |
|  | (0.015) | (0.015) | (0.014) | (0.014) | (0.010) | (0.010) | (0.010) | (0.010) |  |
| Person Mean-Centered Daily Pollutant level | 0.000 | 0.000 | **0.007^c^** | **0.007^c^** | -0.000 | -0.000 | -0.000 | -0.001 |  |
|  | (0.001) | (0.001) | (0.003) | (0.003) | (0.000) | (0.000) | (0.002) | (0.002) |  |
| Person Average Pollutant Level | -0.004 | -0.002 | 0.052 | 0.048 | -0.002 | -0.001 | 0.018 | 0.013 |  |
|  | (0.002) | (0.002) | (0.036) | (0.032) | (0.002) | (0.002) | (0.030) | (0.028) |  |
| Physical Symptoms Each Year |  | **0.231^a^** |  | **0.233^a^** |  | **0.163^a^** |  | **0.164^a^** |  |
|  |  | (0.023) |  | (0.023) |  | (0.020) |  | (0.020) |  |
| Daily Pollutant level X Physical Symptoms |  | -0.001 |  | 0.003 |  | -0.000 |  | -0.002 |  |
|  |  | (0.001) |  | (0.003) |  | (0.000) |  | (0.002) |  |
| Constant | **1.854^a^** | **1.793^a^** | **1.561^a^** | **1.572^a^** | **1.555^a^** | **1.514^a^** | **1.417^a^** | **1.428^a^** |  |
|  | (0.101) | (0.092) | (0.097) | (0.088) | (0.086) | (0.080) | (0.082) | (0.077) |  |
| Observations | 9,513 | 9,401 | 9,513 | 9,401 | 9,525 | 9,413 | 9,525 | 9,413 |  |

*Table S3*

Multilevel regressions predicting fatigue and emotional distress the same day as SO_2_ and O_3_ levels.

|  | Daily Fatigue Same Day | | | | | | | | Daily Emotional Distress Same Day | | | | | | | | | | |
| --- | --- | --- | --- | --- | --- | --- | --- | --- | --- | --- | --- | --- | --- | --- | --- | --- | --- | --- | --- |
|  | NO_2_ -> Daily Fatigue | | CO -> Daily Fatigue | | O_3_ -> Daily Fatigue | | SO_2_ -> Daily Fatigue | | NO_2_ -> Daily Emotional Distress | | CO -> Daily Emotional Distress | | | O_3_ -> Daily Emotional Distress | | | SO_2_ -> Daily Emotional Distress | | |
|  | Model 1 | Model 2 | Model 1 | Model 2 | Model 1 | Model 2 | Model 1 | Model 2 | Model 1 | Model 2 | Model 1 | Model 2 | Model 1 | | Model 2 | Model 1 | | Model 2 |  |
|  | *B* | *B* | *B* | *B* | *B* | *B* | *B* | *B* | *B* | *B* | *B* | *B* | *B* | | *B* | *B* | | *B* |  |
|  | *(SE)* | *(SE)* | *(SE)* | *(SE)* | *(SE)* | *(SE)* | *(SE)* | *(SE)* | *(SE)* | *(SE)* | *(SE)* | *(SE)* | *(SE)* | | *(SE)* | *(SE)* | | *(SE)* |  |
| Weekday | 0.070*** | 0.068*** | 0.084*** | 0.084*** | 0.080*** | 0.079*** | 0.083*** | 0.082*** | 0.108*** | 0.107*** | 0.112*** | 0.113*** | 0.105*** | | 0.106*** | 0.114*** | | 0.114*** |  |
|  | (0.018) | (0.018) | (0.016) | (0.016) | (0.017) | (0.017) | (0.016) | (0.016) | (0.016) | (0.016) | (0.015) | (0.015) | (0.015) | | (0.015) | (0.014) | | (0.014) |  |
| Person Mean-Centered Daily Pollutant level | 0.003 | 0.003* | -0.001 | -0.001 | -0.000 | -0.000 | 0.004 | 0.003 | 0.001 | 0.001 | 0.003 | 0.003 | -0.001 | | -0.001 | -0.004 | | -0.005 |  |
|  | (0.001) | (0.001) | (0.004) | (0.004) | (0.001) | (0.001) | (0.004) | (0.004) | (0.001) | (0.001) | (0.004) | (0.004) | (0.001) | | (0.001) | (0.003) | | (0.003) |  |
| Person Average Pollutant Level | 0.010* | 0.008 | 0.026* | 0.018 | -0.005* | -0.004 | 0.053 | 0.049 | 0.007 | 0.004 | 0.019 | 0.011 | -0.004 | | -0.003 | 0.024 | | 0.017 |  |
|  | (0.005) | (0.005) | (0.012) | (0.011) | (0.003) | (0.002) | (0.040) | (0.036) | (0.005) | (0.005) | (0.013) | (0.012) | (0.003) | | (0.003) | (0.044) | | (0.041) |  |
| Temperature | 0.002 | 0.002 | 0.004 | 0.004 | 0.004 | 0.004 | 0.002 | 0.002 | 0.001 | 0.001 | 0.002 | 0.002 | 0.004 | | 0.003 | 0.002 | | 0.002 |  |
|  | (0.002) | (0.002) | (0.002) | (0.002) | (0.002) | (0.002) | (0.002) | (0.002) | (0.002) | (0.002) | (0.002) | (0.002) | (0.002) | | (0.002) | (0.002) | | (0.002) |  |
| Humidity | -0.002* | -0.002* | -0.002** | -0.002** | -0.002** | -0.002** | -0.002** | -0.002** | -0.001 | -0.001 | -0.001 | -0.001 | -0.001 | | -0.001 | -0.001 | | -0.001 |  |
|  | (0.001) | (0.001) | (0.001) | (0.001) | (0.001) | (0.001) | (0.001) | (0.001) | (0.001) | (0.001) | (0.001) | (0.001) | (0.001) | | (0.001) | (0.001) | | (0.001) |  |
| Physical Symptoms Each Year |  | 0.581*** |  | 0.580*** |  | 0.581*** |  | 0.586*** |  | 0.533*** |  | 0.532*** |  | | 0.532*** |  | | 0.536*** |  |
|  |  | (0.059) |  | (0.059) |  | (0.059) |  | (0.059) |  | (0.065) |  | (0.065) |  | | (0.065) |  | | (0.065) |  |
| Daily Pollutant level X Physical Symptoms |  | 0.003*** |  | 0.010** |  | -0.001 |  | 0.003 |  | 0.003** |  | 0.009** |  | | -0.001 |  | | -0.002 |  |
|  |  | (0.001) |  | (0.003) |  | (0.001) |  | (0.003) |  | (0.001) |  | (0.003) |  | | (0.001) |  | | (0.003) |  |
| Constant | -0.334 | -1.177*** | -0.304 | -1.178*** | 0.065 | -0.924*** | -0.159 | -1.101*** | -0.311 | -1.048*** | -0.258 | -1.033*** | -0.052 | | -0.936*** | -0.207 | | -1.038*** |  |
|  | (0.248) | (0.254) | (0.192) | (0.208) | (0.166) | (0.188) | (0.187) | (0.203) | (0.242) | (0.254) | (0.181) | (0.203) | (0.166) | | (0.195) | (0.184) | | (0.207) |  |
| Observations | 9,499 | 9,387 | 9,499 | 9,387 | 9,499 | 9,387 | 9,499 | 9,387 | 9,511 | 9,399 | 9,511 | 9,399 | 9,511 | | 9,399 | 9,511 | | 9,399 |  |

*Table S4*

Daily and average level associations between air pollutants (NO_2_, CO, O_3_ and SO_2_) predicting adolescents fatigue and distress, using three level hierarchical linear regression models that nested days within years within individuals, when controlling for daily temperature and relative humidity levels.

*Note:* Standard errors in parentheses. *** p<0.001, ** p<0.01, * p<0.05

*Table S5*

|  | Next day fatigue | | | | | | | | Next day emotional distress | | | | | | | |
| --- | --- | --- | --- | --- | --- | --- | --- | --- | --- | --- | --- | --- | --- | --- | --- | --- |
|  | NO_2_ ->Next Day Fatigue | | CO ->Next Day Fatigue | | O_3_ ->Next Day Fatigue | | SO_2_ ->Next Day Fatigue | | NO_2_ ->Next Day emotional distress | | CO ->Next Day emotional distress | | O_3_ ->Next Day emotional distress | | SO_2_ ->Next Day emotional distress | |
|  | Model 1 | Model 2 | Model 1 | Model 2 | Model 1 | Model 2 | Model 1 | Model 2 | Model 1 | Model 2 | Model 1 | Model 2 | Model 1 | Model 2 | Model 1 | Model 2 |
|  | *B* | *B* | *B* | *B* | *B* | *B* | *B* | *B* | *B* | *B* | *B* | *B* | *B* | *B* | *B* | *B* |
|  | *(SE)* | *(SE)* | *(SE)* | *(SE)* | *(SE)* | *(SE)* | *(SE)* | *(SE)* | *(SE)* | *(SE)* | *(SE)* | *(SE)* | *(SE)* | *(SE)* | *(SE)* | *(SE)* |
| Weekday | 0.009 | 0.009 | 0.018 | 0.019 | 0.016 | 0.018 | 0.020 | 0.021 | 0.015 | 0.015 | 0.015 | 0.016 | 0.016 | 0.016 | 0.015 | 0.017 |
|  | (0.016) | (0.016) | (0.015) | (0.015) | (0.015) | (0.015) | (0.015) | (0.015) | (0.011) | (0.011) | (0.010) | (0.010) | (0.010) | (0.010) | (0.010) | (0.010) |
| Same day levels of outcome | **0.180^a^** | **0.176^a^** | **0.180^a^** | **0.177^a^** | **0.181^a^** | **0.178^a^** | **0.180^a^** | **0.177^a^** | **0.202^a^** | **0.197^a^** | **0.202^a^** | **0.196^a^** | **0.202^a^** | **0.197^a^** | **0.203^a^** | **0.197^a^** |
|  | (0.011) | (0.011) | (0.011) | (0.011) | (0.011) | (0.011) | (0.011) | (0.011) | (0.011) | (0.011) | (0.011) | (0.011) | (0.011) | (0.011) | (0.011) | (0.011) |
| Person Mean-Centered daily Pollutant level | **0.002^c^** | **0.002^c^** | 0.005 | 0.005 | -0.001 | -0.001 | 0.004 | 0.004 | 0.000 | 0.000 | 0.001 | 0.001 | -0.000 | -0.000 | 0.002 | 0.001 |
|  | (0.001) | (0.001) | (0.003) | (0.003) | (0.001) | (0.001) | (0.003) | (0.003) | (0.001) | (0.001) | (0.002) | (0.002) | (0.000) | (0.000) | (0.002) | (0.002) |
| Person Average Pollutant Level | **0.010^c^** | 0.008 | **0.023^c^** | 0.016 | -0.004 | -0.003 | 0.060 | 0.056 | 0.005 | 0.004 | 0.014 | 0.009 | -0.002 | -0.002 | 0.033 | 0.029 |
|  | (0.004) | (0.004) | (0.010) | (0.009) | (0.002) | (0.002) | (0.036) | (0.033) | (0.004) | (0.003) | (0.009) | (0.008) | (0.002) | (0.002) | (0.031) | (0.029) |
| Physical Symptoms Each Year |  | **0.198^a^** |  | **0.198^a^** |  | **0.199^a^** |  | **0.200^a^** |  | **0.136^a^** |  | **0.136^a^** |  | **0.136^a^** |  | **0.137^a^** |
|  |  | (0.023) |  | (0.023) |  | (0.023) |  | (0.023) |  | (0.019) |  | (0.019) |  | (0.019) |  | (0.019) |
| Daily Pollutant level X Physical Symptoms |  | 0.001 |  | 0.005 |  | -0.001 |  | -0.000 |  | 0.001 |  | **0.005^c^** |  | -0.000 |  | 0.003 |
|  |  | (0.001) |  | (0.003) |  | (0.001) |  | (0.003) |  | (0.001) |  | (0.002) |  | (0.000) |  | (0.002) |
| Constant | **1.433^a^** | **1.495^a^** | **1.562^a^** | **1.603^a^** | **1.869^a^** | **1.817^a^** | **1.549^a^** | **1.562^a^** | **1.349^a^** | **1.389^a^** | **1.406^a^** | **1.434^a^** | **1.592^a^** | **1.560^a^** | **1.406^a^** | **1.416^a^** |
|  | (0.127) | (0.117) | (0.070) | (0.065) | (0.102) | (0.093) | (0.099) | (0.090) | (0.109) | (0.103) | (0.060) | (0.057) | (0.087) | (0.082) | (0.084) | (0.079) |
| Observations | 8,547 | 8,447 | 8,547 | 8,447 | 8,547 | 8,447 | 8,547 | 8,447 | 8,558 | 8,458 | 8,558 | 8,458 | 8,558 | 8,458 | 8,558 | 8,458 |

Multilevel regressions predicting fatigue and emotional distress the next day after air pollution

*Note.* Standard errors in parentheses. ^a^ = *p*<0.001, ^b^ = *p*<0.01, ^c^ = *p*<0.05. Significant associations are also bolded.

*Figure S1*

Daily CO levels are positively, marginally related to greater feelings of distress the next day among adolescents who report greater ongoing physical symptoms for the year (+1 *SD* above the mean level of physical symptoms) but not among adolescents who report fewer ongoing physical symptoms for the year (-1 *SD* below the mean level of physical symptoms).


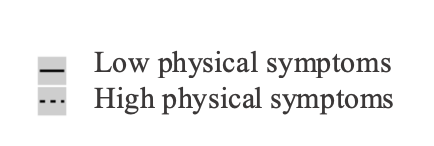

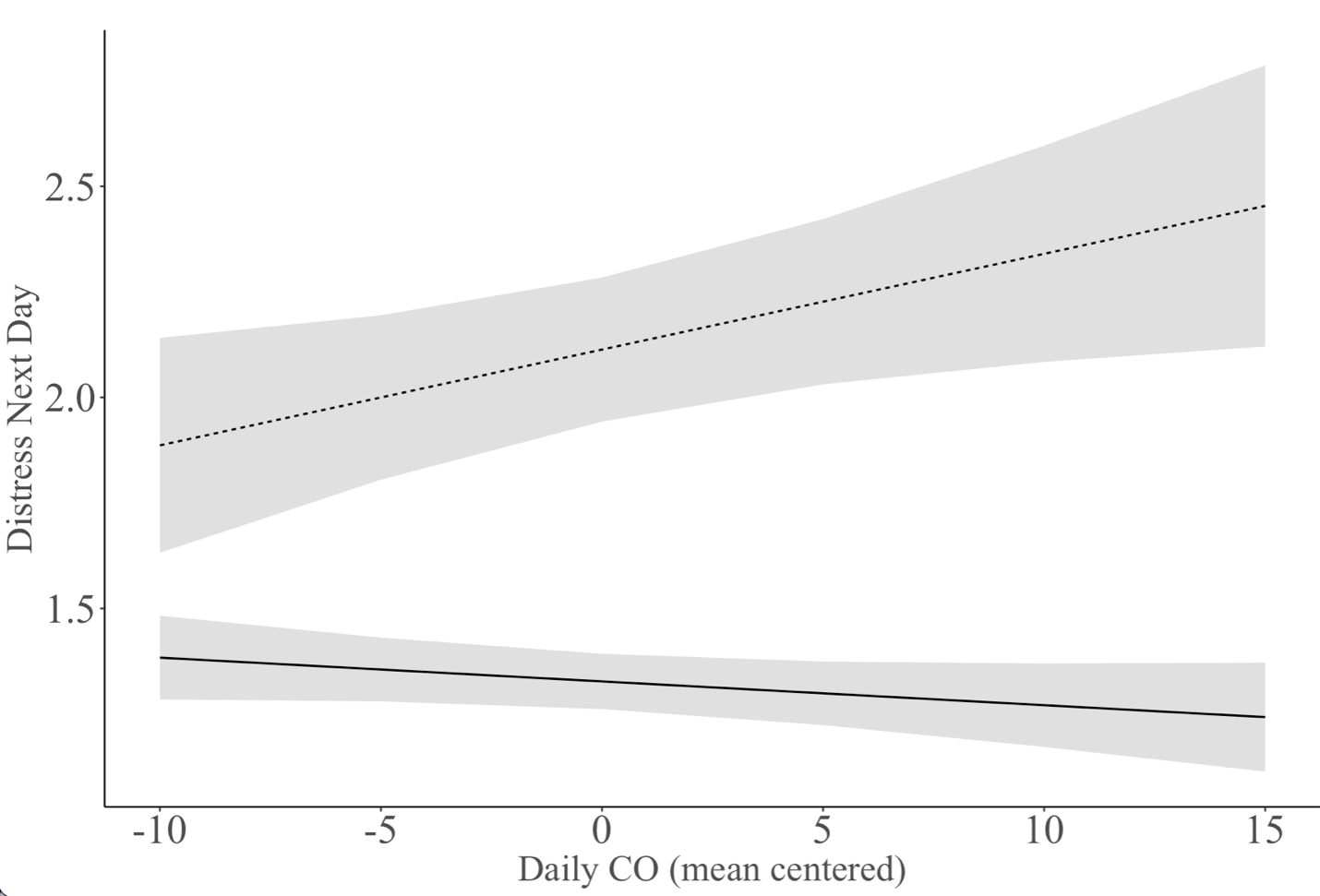


*B* = -0.004, *SE* = 0.003, *p* = .210

*B* = 0.006, *SE* = 0.003, *p* = .061

*Table S6*

Daily and average level associations between air pollutants (PM25 and PM10) predicting adolescents fatigue and distress, using three level hierarchical linear regression models that nested days within years within individuals.

|  | PM10 --> Fatigue | | | | PM10 -->Emotional Distress | | | |
| --- | --- | --- | --- | --- | --- | --- | --- | --- |
|  | PM10 --> Fatigue | | PM10 --> Fatigue | | PM10 --> Distress | | PM10 --> Distress | |
|  | Model 1 | Model 2 | Model 1 | Model 2 | Model 1 | Model 2 | Model 1 | Model 2 |
| Weekday | 0.077*** | 0.075*** | 0.077*** | 0.076*** | 0.078*** | 0.079*** | 0.078*** | 0.078*** |
|  | (0.014) | (0.014) | (0.014) | (0.014) | (0.010) | (0.010) | (0.010) | (0.010) |
| Person Mean-Centered Daily Pollutant level | 0.001 | 0.001 | -0.000 | -0.000 | 0.000 | 0.000 | 0.000 | 0.000 |
|  | (0.001) | (0.001) | (0.001) | (0.001) | (0.000) | (0.000) | (0.000) | (0.000) |
| Person Average Pollutant Level | -0.001 | -0.000 | 0.002 | 0.002 | 0.001 | 0.001 | -0.000 | -0.001 |
|  | (0.004) | (0.004) | (0.004) | (0.004) | (0.004) | (0.003) | (0.004) | (0.003) |
| Physical Symptoms Each Year |  | 0.233*** |  | 0.233*** |  | 0.165*** |  | 0.164*** |
|  |  | (0.023) |  | (0.023) |  | (0.020) |  | (0.020) |
| Daily Pollutant level X Physical Symptoms |  | 0.000 |  | -0.000 |  | 0.001 |  | 0.000 |
|  |  | (0.001) |  | (0.001) |  | (0.000) |  | (0.000) |
| Constant | 1.719*** | 1.707*** | 1.619*** | 1.637*** | 1.444*** | 1.429*** | 1.465*** | 1.488*** |
|  | (0.152) | (0.138) | (0.134) | (0.122) | (0.128) | (0.120) | (0.113) | (0.106) |
| Observations | 9,513 | 9,401 | 9,513 | 9,401 | 9,525 | 9,413 | 9,525 | 9,413 |
| Standard errors in parentheses |  |  |  |  |  |  |  |  |
| *** p<0.001, ** p<0.01, * p<0.05 |  |  |  |  |  |  |  |  |

*Table S7:*

Multilevel regressions predicting fatigue and emotional distress three days after air pollution

|  | Fatigue three days later | | | | | | | | Emotional distress three days later | | | | | | | |
| --- | --- | --- | --- | --- | --- | --- | --- | --- | --- | --- | --- | --- | --- | --- | --- | --- |
|  | NO_2_ -> Daily Fatigue | | CO -> Daily Fatigue | | O_3_ -> Daily Fatigue | | SO_2_ -> Daily Fatigue | | NO_2_ -> Daily Emotional Distress | | CO -> Daily Emotional Distress | | O_3_ -> Daily Emotional Distress | | SO_2_ -> Daily Emotional Distress | |
|  | Model 1 | Model 2 | Model 1 | Model 2 | Model 1 | Model 2 | Model 1 | Model 2 | Model 1 | Model 2 | Model 1 | Model 2 | Model 1 | Model 2 | Model 1 | Model 2 |
|  | *B* | *B* | *B* | *B* | *B* | *B* | *B* | *B* | *B* | *B* | *B* | *B* | *B* | *B* | *B* | *B* |
|  | *(SE)* | *(SE)* | *(SE)* | *(SE)* | *(SE)* | *(SE)* | *(SE)* | *(SE)* | *(SE)* | *(SE)* | *(SE)* | *(SE)* | *(SE)* | *(SE)* | *(SE)* | *(SE)* |
| Weekday | -0.056** | -0.052** | -0.054*** | -0.051** | -0.059*** | -0.057*** | -0.054*** | -0.051** | -0.036** | -0.037** | -0.035** | -0.036** | -0.034** | -0.035** | -0.036** | -0.037** |
|  | (0.017) | (0.017) | (0.016) | (0.016) | (0.017) | (0.017) | (0.016) | (0.016) | (0.012) | (0.012) | (0.011) | (0.011) | (0.012) | (0.012) | (0.011) | (0.011) |
| Same Day levels of outcome | -0.010 | -0.010 | -0.010 | -0.010 | -0.010 | -0.010 | -0.010 | -0.010 | -0.017 | -0.018 | -0.017 | -0.018 | -0.017 | -0.018 | -0.017 | -0.018 |
|  | (0.012) | (0.012) | (0.012) | (0.012) | (0.012) | (0.012) | (0.012) | (0.012) | (0.012) | (0.012) | (0.012) | (0.012) | (0.012) | (0.012) | (0.012) | (0.012) |
| Person Mean-Centered Daily Pollutant level |  | 0.000 | 0.001 | 0.001 | -0.001 | -0.001 | 0.002 | 0.002 | 0.000 | 0.000 | -0.001 | -0.001 | 0.000 | 0.000 | 0.001 | 0.001 |
|  |  | (0.001) | (0.004) | (0.004) | (0.001) | (0.001) | (0.003) | (0.003) | (0.001) | (0.001) | (0.003) | (0.003) | (0.000) | (0.000) | (0.002) | (0.002) |
| Person Average Pollutant Level |  | 0.008 | 0.024* | 0.017 | -0.004 | -0.003 | 0.074 | 0.067 | 0.003 | 0.002 | 0.008 | 0.003 | -0.001 | -0.000 | 0.009 | 0.004 |
|  |  | (0.004) | (0.010) | (0.010) | (0.002) | (0.002) | (0.038) | (0.035) | (0.004) | (0.004) | (0.009) | (0.008) | (0.002) | (0.002) | (0.032) | (0.031) |
| Physical Symptoms Each Year |  | 0.229*** |  | 0.228*** |  | 0.229*** |  | 0.231*** |  | 0.159*** |  | 0.159*** |  | 0.159*** |  | 0.159*** |
|  |  | (0.025) |  | (0.025) |  | (0.025) |  | (0.025) |  | (0.021) |  | (0.021) |  | (0.021) |  | (0.021) |
| Daily Pollutant level X Physical Symptoms |  | -0.002* |  | -0.006 |  | -0.002* |  |  |  | -0.000 |  | 0.001 |  | -0.000 |  |  |
|  |  | (0.001) |  | (0.003) |  | (0.001) |  |  |  | (0.001) |  | (0.002) |  | (0.000) |  |  |
| Constant | 1.436*** | 1.507*** | 1.578*** | 1.622*** | 1.907*** | 1.842*** | 1.541*** | 1.556*** | 1.419*** | 1.472*** | 1.462*** | 1.496*** | 1.569*** | 1.528*** | 1.494*** | 1.506*** |
|  | (0.134) | (0.123) | (0.074) | (0.068) | (0.106) | (0.097) | (0.103) | (0.094) | (0.112) | (0.106) | (0.061) | (0.058) | (0.089) | (0.083) | (0.087) | (0.082) |
| Observations | 6,898 | 6,817 | 6,898 | 6,817 | 6,898 | 6,817 | 6,898 | 6,817 | 6,907 | 6,826 | 6,907 | 6,826 | 6,907 | 6,826 | 6,907 | 6,826 |
| Standard errors in parentheses. *** p<0.001, ** p<0.01, * p<0.05 | | | | | |  |  |  |  |  |  |  |  |  |  |  |
|  |  |  |  |  |  |  |  |  |  |  |  |  |  |  |  |  |

*Figure S2*

Daily NO_2_ levels are positively, marginally related to greater feelings of fatigue three days later among adolescents who report fewer ongoing physical symptoms for the year (+1 *SD* above the mean level of physical symptoms) but not among adolescents who report more ongoing physical symptoms for the year (-1 *SD* below the mean level of physical symptoms), controlling for same day levels of fatigue.


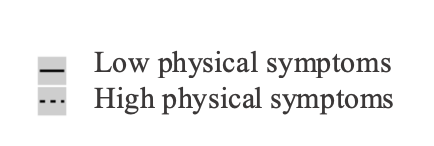

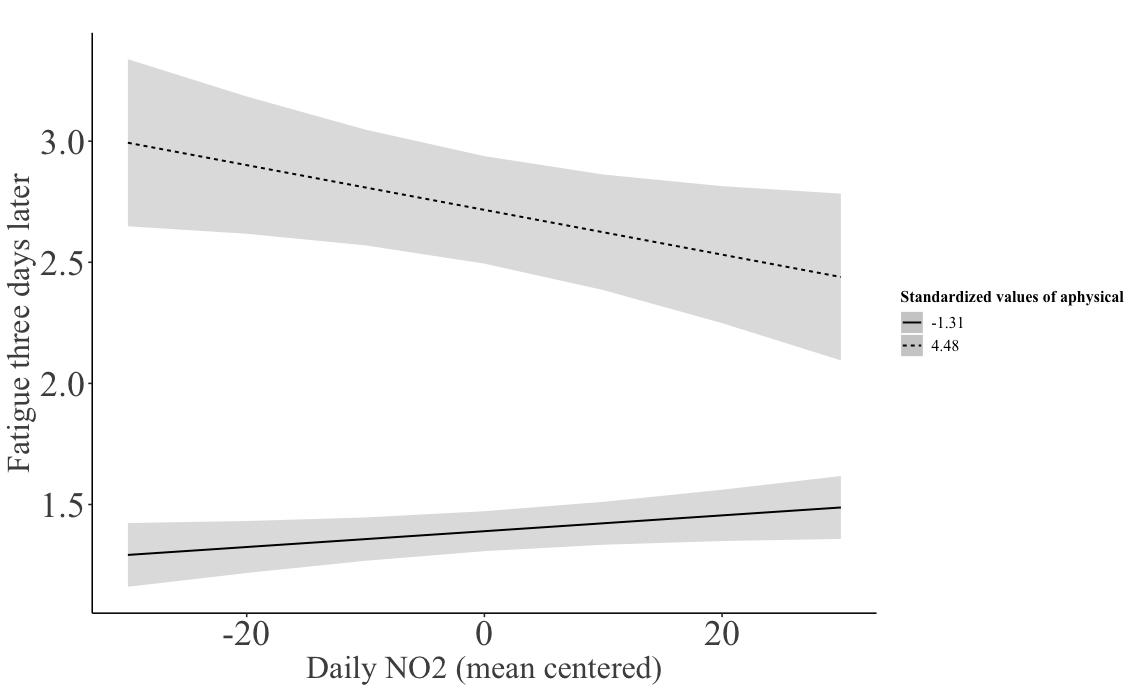


*B* = -0.002, *SE* = 0.001, *p* = .229

*B* = 0.003, *SE* = 0.002, *p* = .084

*Figure S3*

Daily O_3_ levels are negatively related to greater feelings of fatigue three days later among adolescents who report more ongoing physical symptoms for the year (+1 *SD* above the mean level of physical symptoms) but not among adolescents who report fewer ongoing physical symptoms for the year (-1 *SD* below the mean level of physical symptoms), controlling for same day levels of fatigue.


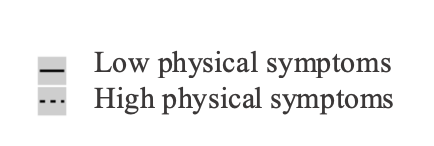

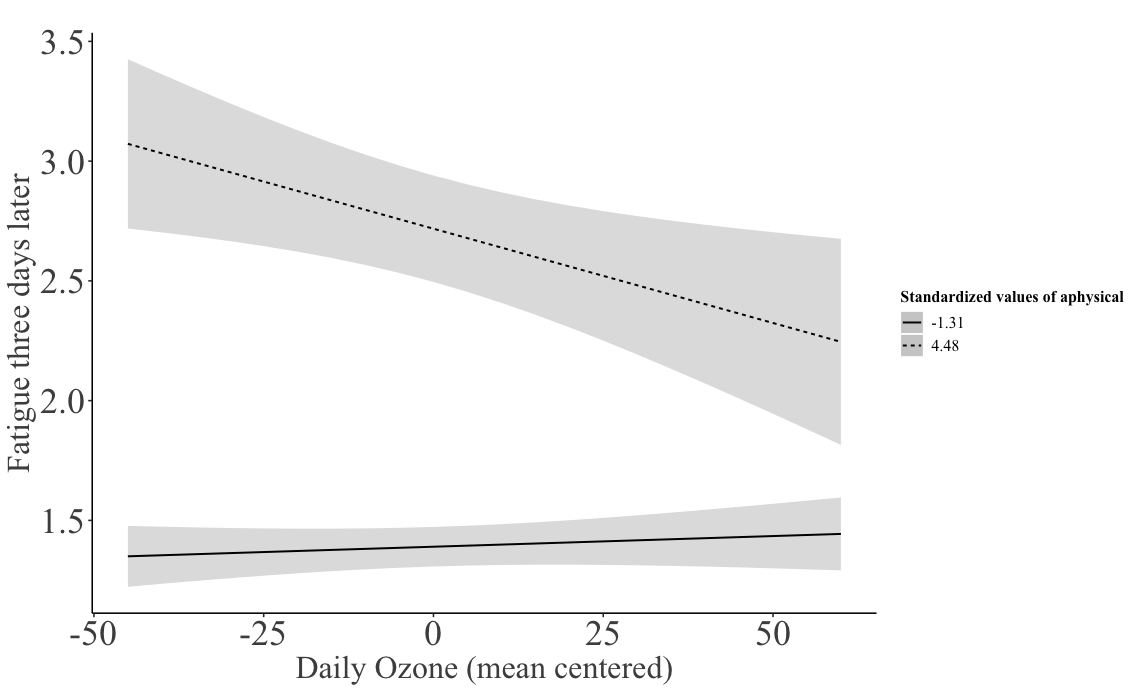


*B* = 0.000, *SE* = 0.001, *p* = .653

*B* = -0.002, *SE* = 0.001, *p* = .009

*Table S8:* Multilevel regressions predicting fatigue and emotional distress five days after air pollution

|  | Daily Fatigue | | | | | | | | Daily Emotional Distress | | | | | | | | | | |
| --- | --- | --- | --- | --- | --- | --- | --- | --- | --- | --- | --- | --- | --- | --- | --- | --- | --- | --- | --- |
|  | NO_2_ -> Daily Fatigue | | CO -> Daily Fatigue | | O_3_ -> Daily Fatigue | | SO_2_ -> Daily Fatigue | | NO_2_ -> Daily Emotional Distress | | CO -> Daily Emotional Distress | | | O_3_ -> Daily Emotional Distress | | | SO_2_ -> Daily Emotional Distress | | |
|  | Model 1 | Model 2 | Model 1 | Model 2 | Model 1 | Model 2 | Model 1 | Model 2 | Model 1 | Model 2 | Model 1 | Model 2 | Model 1 | | Model 2 | Model 1 | | Model 2 |  |
|  | *B* | *B* | *B* | *B* | *B* | *B* | *B* | *B* | *B* | *B* | *B* | *B* | *B* | | *B* | *B* | | *B* |  |
|  | *(SE)* | *(SE)* | *(SE)* | *(SE)* | *(SE)* | *(SE)* | *(SE)* | *(SE)* | *(SE)* | *(SE)* | *(SE)* | *(SE)* | *(SE)* | | *(SE)* | *(SE)* | | *(SE)* |  |
| Weekday | -0.020 | -0.021 | -0.011 | -0.010 | -0.021 | -0.021 | -0.017 | -0.017 | -0.011 | -0.013 | -0.008 | -0.009 | 0.003 | | 0.004 | -0.006 | | -0.006 |  |
|  | (0.019) | (0.019) | (0.018) | (0.019) | (0.018) | (0.018) | (0.018) | (0.018) | (0.014) | (0.014) | (0.013) | (0.013) | (0.013) | | (0.013) | (0.013) | | (0.013) |  |
| Same Day levels of outcome | -0.026* | -0.028* | -0.026* | -0.028* | -0.026* | -0.029* | -0.026* | -0.028* | -0.037** | -0.038** | -0.037** | -0.038** | -0.036* | | -0.037** | -0.036* | | -0.038** |  |
|  | (0.013) | (0.013) | (0.013) | (0.013) | (0.013) | (0.013) | (0.013) | (0.013) | (0.014) | (0.014) | (0.014) | (0.014) | (0.014) | | (0.014) | (0.014) | | (0.014) |  |
| Person Mean-Centered Daily Pollutant level | 0.000 | 0.001 | 0.001 | 0.001 | 0.005 | 0.006 | -0.002 | -0.002 | 0.001 | 0.001 | 0.004 | 0.005 | 0.001* | | 0.002** | 0.001 | | 0.002 |  |
|  | (0.001) | (0.001) | (0.001) | (0.001) | (0.004) | (0.004) | (0.004) | (0.004) | (0.001) | (0.001) | (0.003) | (0.003) | (0.001) | | (0.001) | (0.003) | | (0.003) |  |
| Person Average Pollutant Level | 0.010* | 0.007 | -0.003 | -0.002 | 0.021 | 0.014 | 0.057 | 0.051 | 0.004 | 0.002 | 0.008 | 0.003 | -0.001 | | -0.000 | 0.012 | | 0.008 |  |
|  | (0.005) | (0.004) | (0.002) | (0.002) | (0.011) | (0.010) | (0.040) | (0.037) | (0.004) | (0.004) | (0.009) | (0.009) | (0.002) | | (0.002) | (0.033) | | (0.032) |  |
| Physical Symptoms Each Year |  | 0.230*** |  | 0.231*** |  | 0.229*** |  | 0.231*** |  | 0.151*** |  | 0.151*** |  | | 0.152*** |  | | 0.151*** |  |
|  |  | (0.026) |  | (0.026) |  | (0.026) |  | (0.026) |  | (0.022) |  | (0.022) |  | | (0.022) |  | | (0.022) |  |
| Daily Pollutant level X Physical Symptoms |  | -0.001 |  | 0.001 |  | 0.002 |  | 0.004 |  | 0.000 |  | 0.001 |  | | 0.000 |  | | 0.001 |  |
|  |  | (0.001) |  | (0.001) |  | (0.004) |  | (0.004) |  | (0.001) |  | (0.003) |  | | (0.001) |  | | (0.003) |  |
| Constant | 1.428*** | 1.497*** | 1.836*** | 1.778*** | 1.568*** | 1.610*** | 1.554*** | 1.569*** | 1.392*** | 1.439*** | 1.447*** | 1.476*** | 1.535*** | | 1.498*** | 1.462*** | | 1.473*** |  |
|  | (0.138) | (0.128) | (0.110) | (0.101) | (0.076) | (0.070) | (0.108) | (0.099) | (0.116) | (0.110) | (0.064) | (0.061) | (0.092) | | (0.087) | (0.090) | | (0.085) |  |
| Observations | 5,436 | 5,371 | 5,436 | 5,371 | 5,436 | 5,371 | 5,436 | 5,371 | 5,446 | 5,381 | 5,446 | 5,381 | 5,446 | | 5,381 | 5,446 | | 5,381 |  |
| Standard errors in parentheses. *** p<0.001, ** p<0.01, * p<0.05 | | |  |  |  |  |  |  |  |  |  |  |  | |  |  | |  |  |

*Table S9:* Multilevel regressions predicting fatigue and emotional distress seven days after air pollution

|  | Daily Fatigue 7 days later | | | | | | | | Daily Emotional Distress 7 days later | | | | | | | |
| --- | --- | --- | --- | --- | --- | --- | --- | --- | --- | --- | --- | --- | --- | --- | --- | --- |
|  | NO_2_ -> Daily Fatigue | | CO -> Daily Fatigue | | O_3_ -> Daily Fatigue | | SO_2_ -> Daily Fatigue | | NO_2_ -> Daily Emotional Distress | | CO -> Daily Emotional Distress | | O_3_ -> Daily Emotional Distress | | SO_2_ -> Daily Emotional Distress | |
|  | Model 1 | Model 2 | Model 1 | Model 2 | Model 1 | Model 2 | Model 1 | Model 2 | Model 1 | Model 2 | Model 1 | Model 2 | Model 1 | Model 2 | Model 1 | Model 2 |
|  | *B* | *B* | *B* | *B* | *B* | *B* | *B* | *B* | *B* | *B* | *B* | *B* | *B* | *B* | *B* | *B* |
|  | *(SE)* | *(SE)* | *(SE)* | *(SE)* | *(SE)* | *(SE)* | *(SE)* | *(SE)* | *(SE)* | *(SE)* | *(SE)* | *(SE)* | *(SE)* | *(SE)* | *(SE)* | *(SE)* |
| Weekday | 0.075*** | 0.073*** | 0.069*** | 0.069*** | 0.076*** | 0.078*** | 0.075*** | 0.075*** | 0.051*** | 0.053*** | 0.050*** | 0.053*** | 0.057*** | 0.063*** | 0.053*** | 0.056*** |
|  | (0.021) | (0.021) | -0.02 | -0.02 | (0.020) | (0.020) | (0.020) | (0.020) | (0.015) | (0.015) | (0.014) | (0.014) | (0.015) | (0.015) | (0.014) | (0.014) |
| Same Day levels of outcome | 0.023 | 0.015 | 0.023 | 0.015 | 0.024 | 0.016 | 0.024 | 0.016 | 0.061*** | 0.052** | 0.060*** | 0.051** | 0.062*** | 0.054** | 0.062*** | 0.054** |
|  | (0.015) | (0.015) | -0.015 | -0.015 | (0.015) | (0.015) | (0.015) | (0.015) | (0.016) | (0.016) | (0.016) | (0.016) | (0.016) | (0.016) | (0.016) | (0.016) |
| Person Mean-Centered Daily Pollutant level | -0.000 | 0.000 | 0.009 | 0.010* | 0.000 | 0.000 | -0.001 | 0.001 | 0.000 | 0.001 | 0.005 | 0.006 | 0.001 | 0.001 | 0.003 | 0.004 |
|  | (0.001) | (0.001) | -0.005 | -0.005 | (0.001) | (0.001) | (0.004) | (0.004) | (0.001) | (0.001) | (0.004) | (0.004) | (0.001) | (0.001) | (0.003) | (0.003) |
| Person Average Pollutant Level | 0.010* | 0.008 | 0.024* | 0.018 | -0.004 | -0.003 | 0.067 | 0.061 | 0.003 | 0.002 | 0.008 | 0.004 | -0.001 | -0.001 | 0.016 | 0.011 |
|  | (0.005) | (0.005) | -0.011 | -0.01 | (0.003) | (0.002) | (0.040) | (0.037) | (0.004) | (0.004) | (0.009) | (0.009) | (0.002) | (0.002) | (0.034) | (0.033) |
| Physical Symptoms Each Year |  | 0.220*** |  | 0.219*** |  | 0.222*** |  | 0.223*** |  | 0.146*** |  | 0.146*** |  | 0.147*** |  | 0.148*** |
|  |  | (0.027) |  | -0.027 |  | (0.027) |  | (0.027) |  | (0.023) |  | (0.023) |  | (0.023) |  | (0.023) |
| Daily Pollutant level X Physical Symptoms |  | 0.002 |  | 0.008 |  | -0.001 |  | 0.000 |  | 0.001 |  | 0.003 |  | -0.000 |  | -0.003 |
|  |  | (0.001) |  | -0.005 |  | (0.001) |  | (0.004) |  | (0.001) |  | (0.004) |  | (0.001) |  | (0.003) |
| Constant | 1.332*** | 1.387*** | 1.476*** | 1.510*** | 1.793*** | 1.743*** | 1.452*** | 1.463*** | 1.347*** | 1.380*** | 1.398*** | 1.419*** | 1.503*** | 1.468*** | 1.405*** | 1.413*** |
|  | (0.144) | (0.134) | -0.079 | -0.074 | (0.114) | (0.106) | (0.110) | (0.102) | (0.120) | (0.115) | (0.066) | (0.063) | (0.095) | (0.090) | (0.093) | (0.089) |
| Observations | 4,070 | 4,021 | 4,070 | 4,021 | 4,070 | 4,021 | 4,070 | 4,021 | 4,073 | 4,024 | 4,073 | 4,024 | 4,073 | 4,024 | 4,073 | 4,024 |
| Standard errors in parentheses. *** p<0.001, ** p<0.01, * p<0.05 | | |  |  |  |  |  |  |  |  |  |  |  |  |  |  |
